# The emergence of *chordin-like1* in gnathostomes may have contributed to the evolution of paired appendages

**DOI:** 10.3389/fcell.2025.1649996

**Published:** 2025-08-29

**Authors:** Galina V. Ermakova, Irina V. Meyntser, Nikolai S. Mugue, Vassily A. Lyubetsky, Andrey G. Zaraisky, Andrey V. Bayramov

**Affiliations:** ^1^ Shemyakin-Ovchinnikov Institute of Bioorganic Chemistry, Russian Academy of Sciences, Moscow, Russia; ^2^ Biological Department of the Mosqvarium Center for Oceanography and Marine Biology, Moscow, Russia; ^3^ Department of Molecular Genetics of the Russian Federal Research Institute of Fisheries and Oceanography (VNIRO), Moscow, Russia; ^4^ Koltzov Institute of Developmental Biology, Russian Academy of Sciences, Moscow, Russia; ^5^ Institute for Information Transmission Problems, Russian Academy of Sciences (Kharkevich Institute), Moscow, Russia; ^6^ Department of Regenerative Medicine of the Pirogov Russian National Research Medical University, Moscow, Russia

**Keywords:** chordin-like, shark, sterlet, amphibian, Xenopus, sturgeon, Acipenser, paired fins

## Abstract

Genomic transformations during early vertebrate evolution, including two rounds of whole-genome duplication, laid the groundwork for the emergence of novel morphological features in jawed vertebrates. Among these innovations, paired appendages represent a major evolutionary milestone, whose development and diversification enabled vertebrates to exploit diverse ecological niches in aquatic, terrestrial, and aerial environments. Here, we combined phylogenetic and local genomic synteny analyses to investigate the evolutionary history of *chordin-like* homologs in vertebrates. Our results indicate that *chordin-like1* first appeared in jawed vertebrates, suggesting a possible link between its origin and the emergence of paired appendages. To explore this hypothesis, we examined *chordin-like1* expression in representatives of basal jawed vertebrate lineages - cartilaginous fishes (grey catshark, *Chiloscyllium griseum*) and sturgeons (sterlet, *Acipenser ruthenus*). We further assessed the expression and functional properties of the *chordin-like1* ortholog in the African clawed frog (*Xenopus laevis*), a representative terrestrial vertebrate with limb morphology that markedly differs from the fins of basal gnathostomes. Together with published data, our findings support a potential role for chordin-like1 in the evolution of paired appendages. In particular, *chordin-like1* may have contributed to the development of the metapterygial element and its derivatives, which formed the structural basis for the evolution of tetrapod limbs.

## Introduction

Vertebrates represent one of the most evolutionarily successful groups of living organisms, having colonized a wide range of ecological niches and exhibiting a remarkable diversity of life strategies (Pascual-Anaya et al., 2022). At early stages of their evolution, ancestral vertebrates acquired a number of morphological innovations that enabled more active lifestyles, spatial mobility, and efficient predation (Striedter and Northcutt, 2020). Among these key innovations are paired appendages - initially in the form of fins in fish and later transformed into limbs in terrestrial vertebrates - as well as the jaw apparatus, derived from the branchial arches (Gillis et al., 2013; Gai and Zhu, 2012).

Among extant vertebrates, paired appendages are a unique feature of gnathostomes (jawed vertebrates) (Coates, 2003; Freitas et al., 2014). Although paired appendages have been identified in fossil jawless vertebrates, current data on their endoskeletal structure remain incomplete, making it difficult to establish homology with the appendages of living vertebrates (Coates, 2003; Enny et al., 2020; Bayramov et al., 2024). The origin of paired appendages has been a subject of continuous scientific interest since the 19th century. Several hypotheses have been proposed, among which the most influential are the lateral fin-fold theory - proposed by Thacher (1877), Mivart (1879), and F. Balfour (1881) - and the gill arch hypothesis, also known as Gegenbaur’s archipterygium theory (Thacher, 1877; Mivart, 1879; Balfour, 1881; Gegenbaur, 1878; Sleight and Gillis, 2020). Both theories were originally based on the embryonic anatomy of cartilaginous fishes.

The gill arch theory initially stemmed from the morphological and positional similarities between branchial arches and the pectoral girdle (Diogo, 2020). Later studies provided additional support through the identification of shared expression patterns of key regulatory genes such as *Shh* and *Fgf8* (Gillis et al., 2009), as well as a common mesodermal cell population involved in the development of both the posterior gill arches and the pectoral girdle (Sleight and Gillis, 2020). However, these arguments remain contentious, as *Shh* and *Fgf8* are also expressed in other structures, including unpaired fins (Freitas et al., 2006; Dahn et al., 2007). Moreover, paleontological evidence suggests that the branchial arch structure of modern sharks is likely secondary, as fossil sharks exhibit gill architectures more similar to those of bony fishes (Pradel et al., 2014). The lack of transitional forms in the fossil record that would support the transformation of gill arches into paired limbs remains an unresolved issue (Pieretti et al., 2015).

The lateral fin-fold theory is based on structural similarities between unpaired and paired fins, and on the observation by Balfour of ectodermal thickenings along the lateral body wall in shark embryos (Balfour, 1881). Further support came from the identification of lateral “stripes of competency” for appendage formation in gnathostomes (Yonei-Tamura et al., 2008). Nonetheless, the theory lacks direct embryological and paleontological evidence, despite some reported instances of ventrolateral folds in fossil forms (Gai et al., 2022).

The observed similarities in the activity of *HoxD* and *Tbx* genes, as well as in the Fgf-Shh regulatory circuit, between paired and unpaired fins have led to the hypothesis that the developmental mechanisms of gnathostome appendages first emerged during the evolution of the more ancient unpaired fins and were subsequently co-opted during the evolution of paired fins (Freitas et al., 2006; Letelier et al., 2021; Hawkins et al., 2022).

To date, none of the existing hypotheses provides a definitive explanation for the evolutionary origin of paired appendages in vertebrates. This underscores the importance of generating new data on the developmental mechanisms of appendage formation, particularly in evolutionarily basal gnathostome lineages.

Due to their morphological and phylogenetic characteristics, the fins of cartilaginous and sturgeon fishes are considered a fundamental model for studying paired appendages in gnathostomes (Ahn and Ho, 2008; Zhang et al., 2012). The fin skeleton in these groups consists of two main components: endoskeletal elements (basalia and radialia) and dermal fin rays. The proximally located basal elements include the propterygium, mesopterygium and metapterygium. Among these, the metapterygium, along with its associated radial elements, served as the key structural basis for the subsequent evolutionary development of tetrapod limbs (Cass et al., 2021). In contrast, in Teleostei, the metapterygium has been lost during the course of evolutionary specialization (Tanaka et al., 2022). The basal fin structure in Teleostei consists only of residual propterygium and mesopterygium elements and radialia - or, according to some researchers, exclusively of radialia divided into proximal and distal components (Yano et al., 2012; Enny et al., 2020; Hawkins et al., 2021). The fin lobe in Teleostei is primarily composed of well-developed dermal fin rays, which lack homologs in the limb skeleton (Yano et al., 2012; Cass et al., 2021). These findings suggest that the fins of modern bony fishes and the tetrapod limbs represent two distinct evolutionary trajectories involving reduction and subsequent specialization of an ancestral appendage (Hawkins et al., 2021; Thompson et al., 2021). Among extant vertebrates, the fin structures of modern sharks and sturgeons most closely resemble the ancestral condition. The primitiveness of shark fin morphology is further supported by studies on the origin of their musculature, which develops through direct extension of embryonic myotomes, whereas in bony fishes and tetrapods it arises via migration of *lbx1*-positive mesenchymal myoblast precursors (Cole and Currie, 2007).

According to current understanding, the initiation of paired appendage primordia in vertebrate embryos begins with the induction of *wnt2b* and *wnt8c* expression in cells of the corresponding regions of the lateral mesoderm, in response to a local increase in retinoic acid (RA) levels and transient inhibition of the signaling pathway activated by Bone Morphogenetic Proteins (BMPs) (Mercader et al., 2006; Christen et al., 2012; Feneck and Logan, 2020). *Wnt2b* and *wnt8c* factors activate the expression of *fgf10* in the presumptive limb bud mesenchyme, which in turn induces the formation of a specialized signaling center, the apical ectodermal ridge (AER), in the overlying ectoderm (Kawakami et al., 2001; Mercader, 2007). The AER is a thickened ectodermal structure located at the distal tip of the growing limb or fin bud (Fernandez-Teran and Ros, 2008; Casanova et al., 2011). Among the most critical factors secreted by the AER is *fgf8*, which induces the formation of another signaling center, the zone of polarizing activity (ZPA), in the posterior mesenchyme of the developing bud (Boulet et al., 2004; Dealy et al., 1997; Jin et al., 2019; Sun et al., 2002). The ZPA is composed of a group of mesenchymal cells that produce the morphogen Sonic Hedgehog (*Shh*) (Riddle et al., 1993; Tulenko et al., 2017; Tickle and Towers, 2017). The interaction between *Fgf* and *Shh* signaling establishes a posterior-to-anterior gradient of *Shh*, which is essential for the patterning of the limb bud along the anterior-posterior axis (Riddle et al., 1993; Crossley et al., 1996).

As limb development progresses, the *Fgf8/Shh* signaling module interacts with another pathway, the *BMP/Gremlin* module, which jointly regulates proximodistal and dorsoventral patterning, outgrowth of the limb bud and digit formation (Zeller et al., 2009; Capdevila et al., 1999; Raspopovic et al., 2014; Cass et al., 2021). BMP signaling also plays an important role in dorsoventral patterning of the limb (Pizette et al., 2001). During limb bud development, *BMP2*, *BMP4*, and *BMP7* are expressed in the AER, while *BMP2* is also expressed in the posterior mesenchymal domain. The BMP antagonist *gremlin* is expressed in the central region of the bud, particularly in cells of the dorsal and ventral mesenchyme (Capdevila et al., 1999).

In addition to *gremlin*, early stages of limb bud development in chick and mouse embryos also show high levels of expression of *chordin-like1*, which encodes another secreted BMP antagonist (Allen et al., 2013; Coffinier et al., 2001; Nakayama et al., 2001). However, unlike *gremlin*, the specific role of *chordin-like1* in BMP signal regulation during early limb development remains poorly understood, current evidence mainly implicates its function in digit formation at later stages (Allen et al., 2013).

Chordin-like1 is a homolog of *Chordin*, a famous BMP antagonist that plays a key role in establishing the dorsoventral gradient of free BMP4 essential for body axis formation (Plouhinec et al., 2013; De Robertis and Moriyama, 2016; De Robertis et al., 2017). A characteristic feature of Chordin and its related proteins is the presence of conserved cysteine-rich (CR) domains that mediate BMP binding. *Chordin* contains four CR domains arranged in a specific pattern - one near the N-terminal and three near the C-terminal (Troilo et al., 2014). Functional studies have shown that CR1 and CR3 are especially critical for BMP binding (Larraín et al., 2000). Chordin-like proteins typically contain three CR domains, which similarly confer the ability to bind BMP ligands (Nakayama et al., 2001). Homologs of Chordin and Chordin-like proteins have been identified in a wide range of invertebrates (DuBuc et al., 2019).

In vertebrates, *Chordin-like* genes - previously also referred to as *neuralin* or *ventroptin* - have been identified in fish (*Danio*), birds (*Gallus*), and mammals (*Muridae*) (Branam et al., 2010; Coffinier et al., 2001; Nakayama et al., 2001; Nakayama et al., 2004; Sakuta et al., 2001; Allen et al., 2013). In early *Danio* embryos, *chordin-like* is expressed more uniformly compared to the sharply graded expression of *chordin* (Branam et al., 2010). *Chordin-like* expression has been reported in the limb buds of chick and mouse embryos (Allen et al., 2013; Coffinier et al., 2001; Nakayama et al., 2001).

In recent years, there has been growing evidence that gene emergence and loss could play an important role in a wide variety of macroevolutionary transformations (Ivanova et al., 2013; Ivanova et al., 2015; Bayramov et al., 2016; Ivanova et al., 2018; Korotkova et al., 2019; Lyubetsky et al., 2023; Zaraisky et al., 2024). Assuming this, we hypothesized that the emergence of the *chordin-like1* gene may have played a significant role in the evolution of paired appendages in gnathostomes. To test this hypothesis, we conducted phylogenetic analyses and examined local genomic synteny of *chordin-like* genes across vertebrates. Our analysis revealed that most vertebrates possess two paralogs: *chordin-like1* and *chordin-like2*. Notably, *chordin-like1* is absent in jawless vertebrates and invertebrate chordates and appears to have emerged specifically in the gnathostome lineage. Since paired appendages also first arise in gnathostomes, we investigated *chordin-like1* expression in the fin buds of basal gnathostome representatives: the catshark (*Chiloscyllium griseum*, representative of cartilaginous fish) and sterlet (*Acipenser ruthenus*, a representative of chondrostean). Additionally, we studied the expression and functional properties of *chordin-like1* in the African clawed frog (*Xenopus laevis*), a representative of terrestrial vertebrates whose limb structure differs significantly from that of basal gnathostomes. Our findings suggest that the emergence of *chordin-like1* may have been among the genetic innovations contributing to the evolution of paired appendages in gnathostomes, particularly by influencing the development of the metapterygial element and its derivatives, which constitute the foundation of tetrapod limb architecture.

## Results

### Phylogenetic and local genomic synteny analysis of *Chordin-like* genes in vertebrates

To investigate the phylogenetic relationships of *Chordin-like* genes in vertebrates, we performed a comprehensive search for Chordin-like homologs in available databases, followed by phylogenetic analysis of the encoded protein sequences, as well as an assessment of local genomic synteny (i.e., the arrangement of neighboring genes) of *Chordin-like* loci in vertebrates, invertebrate chordates, and some representatives of protostomes.

**FIGURE 1:**
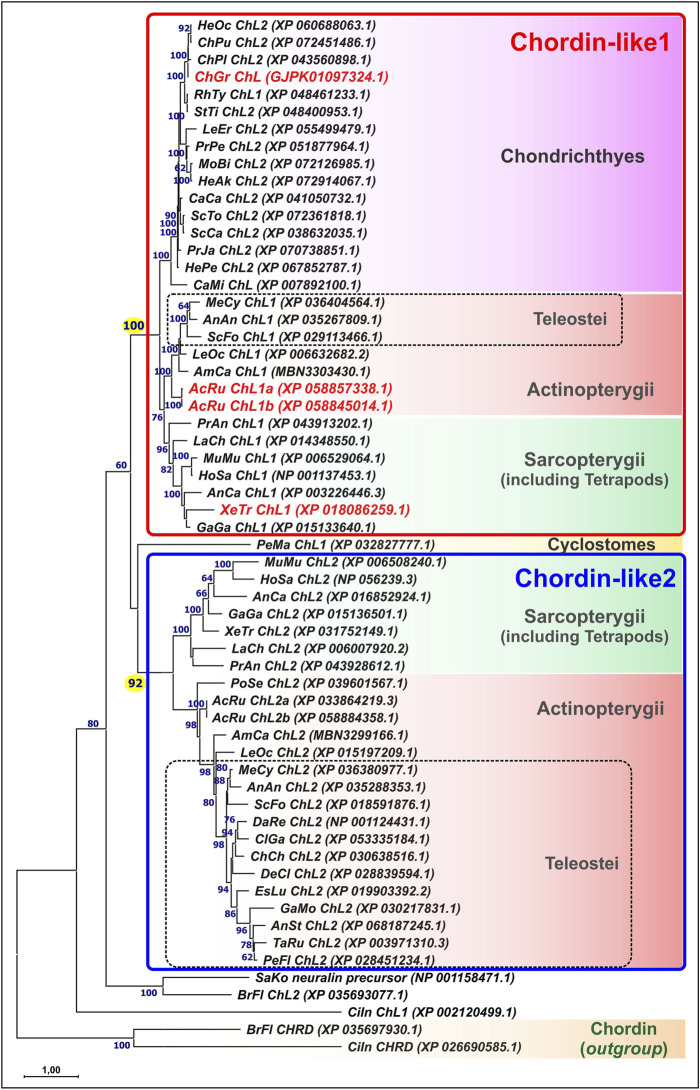
Maximum likelihood (ML) phylogenetic tree of Chordin-like proteins in chordates. Chordin-like proteins from *C. griseum*, *A. ruthenus*, and *Xenopus tropicalis* are highlighted in red. Bootstrap support values greater than 60 are indicated. Branch lengths represent the number of substitutions per site, as shown by the scale bar. Most analyzed representatives of Gnathostomes possess two paralogs, Chordin-like1 and Chordin-like2, which can be confidently distinguished, with bootstrap support values of 100 and 92, respectively. In Chondrichthyes, only Chordin-like1 is present. In the basally divergent lineages of Teleostei (Elopomorpha and Osteoglossomorpha), both Chordin-like paralogs are retained, whereas in the majority of the examined Teleostei species, only Chordin-like2 is detected. AcRu–*Acipenser ruthenus*, AmCa–*Amia calva*, AnAn - *Anguilla anguilla*, AnCa–*Anolis carolinensis,* AnSt - *Antennarius striatus*, BrFl–*Branchiostoma floridae*, CaCa - *Carcharodon carcharias*, CaMi - *Callorhinchus milii*, ChCh - *Chanos chanos*, ChGr - *Chiloscyllium griseum*, ChPu - *Chiloscyllium punctatum*, ChPl–*Chiloscyllium plagiosum*, CiIn–*Ciona intestinalis*, ClGa - *Clarias gariepinus*, DaRe–*Danio rerio*, DeCl - *Denticeps clupeoides*, EsLu - *Esox lucius*, GaGa - *Gallus gallus*, GaMo - *Gadus morhua*, HeAk - *Hemitrygon akajei*, HeOc - *Hemiscyllium ocellatum*, HePe - *Heptranchias perlo*, HoSa–*Homo sapiens*, LaCh–*Latimeria chalumnae*, LeEr–*Leucoraja erinacea,* LeOc–*Lepisosteus oculatus*, MeCy–*Megalops cyprinoides*, MoBu - *Mobula birostris*, MuMu–*Mus musculus*, PeFl - *Perca flavescens*, PeMa–*Petromyzon marinus*, PoSe–*Polypterus senegalus*, PrAn–*Protopterus annectens*, PrJa - *Pristiophorus japonicas*, PrPe - *Pristis pectinata*, RhTy - *Rhincodon typus*, SaKo–*Saccoglossus kowalevskii,* ScCa - *Scyliorhinus canicula*, ScFo - *Scleropages formosus*, ScTo - *Scyliorhinus torazame*, StTi - *Stegostoma tigrinum*, TaRu–*Takifugu rubripes*, XeTr–*Xenopus tropicalis*.

Phylogenetic analysis of Chordin-like protein sequences (Figure 1) revealed that the genomes of most vertebrate species contain two paralogous *Chordin-like* genes. Gene nomenclature was adopted according to Nakayama et al. (2001); Nakayama et al. (2004).

The Chordin-like1 and Chordin-like2 protein clusters were found to be strongly supported, with bootstrap values of 100 in a maximum likelihood (ML) tree constructed using optimal substitution models in MEGA11. This robust clustering was observed for the majority of vertebrate sequences, with the exception of genes from evolutionarily basal chordate lineages: tunicates (*Ciona*), amphioxus (*Branchiostoma*), and hemichordates (*Saccoglossus*), as well as from the sea lamprey *Petromyzon marinus*, a representative of jawless vertebrates.

Chordin-like proteins are clearly distinguishable from the structurally similar Chordin protein, which also contains cysteine-rich (CR, cis-rich) domains and was included in the analysis as an outgroup. Analysis of the distribution of conserved cis-rich domains in Chordin-like proteins across different vertebrate groups revealed that most Chordin-like proteins contain three cis-rich domains (Supplementary Figure S1). Exceptions include the Chordin-like1 protein of amphibians, which contains two domains, and the Chordin-like2 protein of coelacanths, which contains only one domain.

**FIGURE 2:**
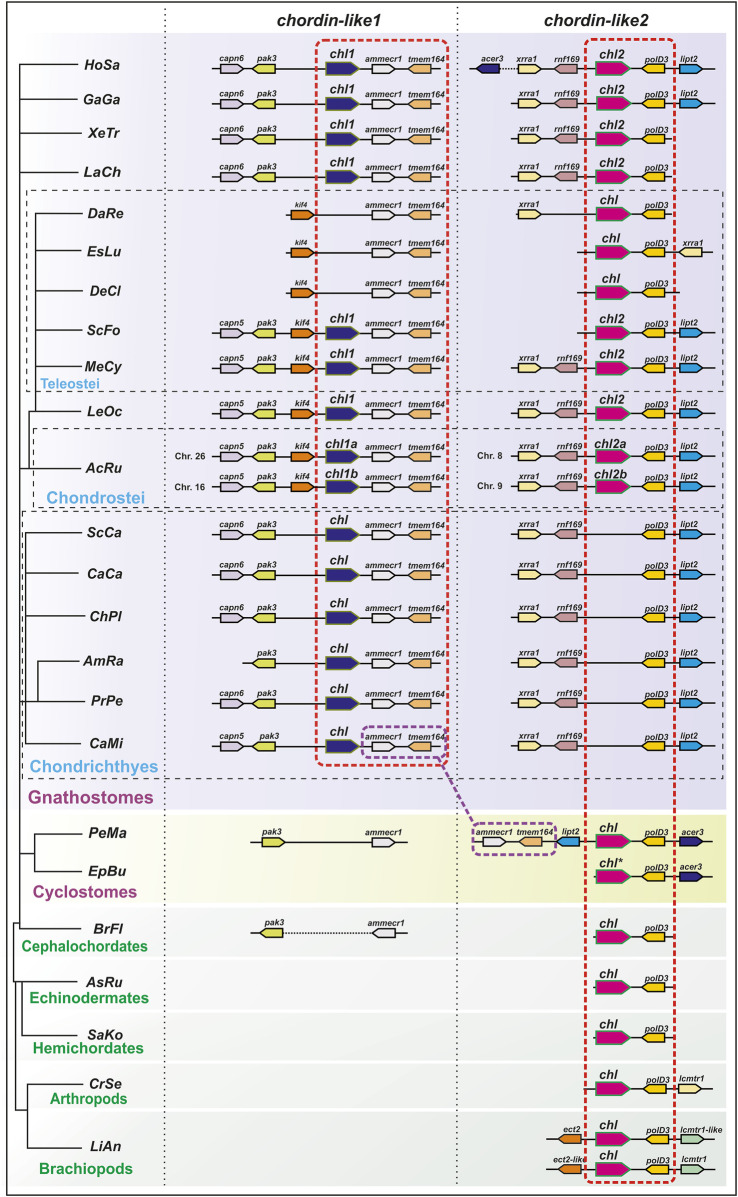
Analysis of local genomic synteny of *chordin-like* genes in chordates and some invertebrates. The *chordin-like2* gene has a neighboring *polD3* gene in the genomes of vertebrates and invertebrates. The *chordin-like1* gene with its characteristic neighboring genes is found only in gnathostomes. The *ammecr* and *tmem164* genes are present in the vicinity of the gnathostome *chordin-like1* genes and the lamprey (*P. marinus*) *chordin-like2* genes. AcRu–*Acipenser ruthenus*, AmRa–*Amblyraja radiate*, AsRu–*Asterias rubens*, BrFl–*Branchiostoma floridae*, CaCa - *Carcharodon carcharias*, CaMi - *Callorhinchus milii*, ChPl–*Chiloscyllium plagiosum*, CrSe - *Cryptotermes secundus*, DaRe–*Danio rerio*, DeCl - *Denticeps clupeoides*, EpBu–*Eptatretus burgeri*, EsLu - *Esox lucius*, GaGa - *Gallus gallus*, HoSa–*Homo sapiens*, LaCh–*Latimeria chalumnae*, LeOc–*Lepisosteus oculatus*, LiAn - *Lingula anatine*, MeCy–*Megalops cyprinoides*, PeMa–*Petromyzon marinus*, PrPe - *Pristis pectinata*, SaKo–*Saccoglossus kowalevskii,* ScCa - *Scyliorhinus canicula*, ScFo - *Scleropages formosus*, XeTr–*Xenopus tropicalis*. * sequence of *E. burgeri chordin-like* (Eptbu0006070.t1) was found in Squalomix sequence server (https://treethinkers.nig.ac.jp/squalomix/blast/).

To confirm the orthology of *chordin-like* genes across different vertebrate lineages, we conducted an analysis of their local genomic synteny in both vertebrate species and selected invertebrate representatives (Figure 2).

The conducted analysis revealed that the synteny between *chordin-like2* and its neighboring gene *polD3* is traceable even at the level of deuterostomes and protostomes (Figure 2). It suggest, that *chordin-like2* gene is ancient and presented in the common ancestor of jawless and jawed vertebrates.

In contrast, based on local genomic synteny analysis, the *chordin-like1* gene appears to have emerged only in jawed vertebrates. Consequently, in most jawed vertebrate groups, two *chordin-like* paralogs are present in the genome: *chordin-like1* and *chordin-like2*. The presence of shared neighboring genes between *chordin-like1* in jawed vertebrates and *chordin-like2* in lamprey–genes *ammecr1* (AMMECR nuclear protein 1) and *tmem164* (transmembrane protein 164) - suggests that *chordin-like1* may have originated via local duplication of *chordin-like2* or as a result of one of the rounds of whole-genome duplication (WGD) early in vertebrate evolution. Additional conserved neighbors of *chordin-like1*, beyond *ammecr1* and *tmem164*, include *pak3* (p21 (RAC1) activated kinase 3) and *capn5/6* (calpain 5 or calpain 6 homologs) in Chondrichthyes, Sarcopterygii, and Tetrapods, as well as *Kif4* (kinesin family member 4) in Actinopterygii (Chondrostei and Neopterygii). The absence of *chordin-like1* in jawless vertebrates and cephalochordates is further supported by the lack of this gene in the genomic regions surrounding *pak3* and *ammecr1* - conserved neighbors of *chordin-like1* in jawed vertebrates. Additionally, *chordin-like1* is missing from the genome of certain teleost species, such as *D. rerio* (Figure 2).

Given the available literature data and considering a possible role of *chordin-like1* emergence in the evolution and development of key morphological innovations in jawed vertebrates, such as paired appendages, gill apparatus, and jaws, we examined its expression during early development in cartilaginous and chondrostean fishes, representing the most evolutionarily basal extant jawed vertebrate groups amenable to laboratory investigation.

In cartilaginous fishes, expression analysis of *chordin-like1* was performed on embryos of the grey catshark (*C*. *griseum*), while in chondrostean, the analysis was conducted on embryos and prelarvae of the sterlet (*A*. *ruthenus*). The expression pattern of *chordin-like1* was assessed using whole-mount *in situ* hybridization (ISH).

### Expression of *chordin-like1* in the grey catshark

Phylogenetic analysis revealed that, unlike most other vertebrate groups, sharks possess only one *chordin-like* gene - *chordin-like1*. We investigated its spatiotemporal expression by ISH in *C. griseum* embryos from the pre-fin bud stage (stage 24) onwards. Developmental staging was based on Ballard et al., 1993.

**FIGURE 3:**
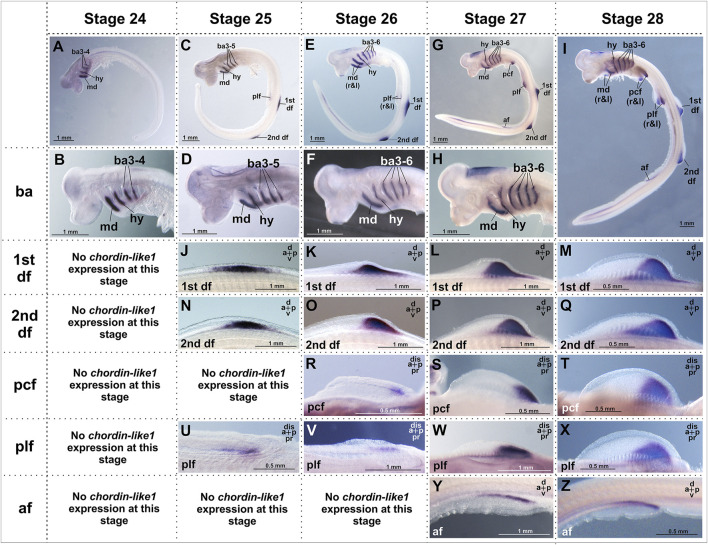
Analysis of the spatial expression of the *chordin-like1* during early developmental stages of the grey catshark *C. griseum*. Staging was performed according to Ballard et al. (1993). At stage 24 **(A, B)**, chordin-like1 expression is observed in the primordia of the mandibular, hyoid, and branchial arches. By stage 25, in addition to expression in these arches **(C, D)**, chordin-like1 expression also appears in the early primordia of the dorsal **(J, N)** and pelvic **(U)** fins. From stage 26 onwards **(E–I)** in addition to branchial arches, chordin-like1 expression is detected in the posterior regions of the developing primordia of both paired (pectoral and pelvic) **(R–T; V–X)** and unpaired dorsal fins **(K–M; O–Q)**. From stage 27 onwards, expression is detected in the anal fin **(Y, Z)**. The photo of the 2nd dorsal fin in panel **(Q)** is the same as that in Figure 4K, and the photo of the pelvic fin in panel **(X)** is the same as that in Figure 4G. a–anterior, af–anal fin, ba–branchial arches, d–dorsal, df–dorsal fin, dis–distal, hy–hyoid arch, md–mandibular arch, p–posterior, pcf–pectoral fin, plf–pelvic fin, pr–proximal.

**FIGURE 4:**
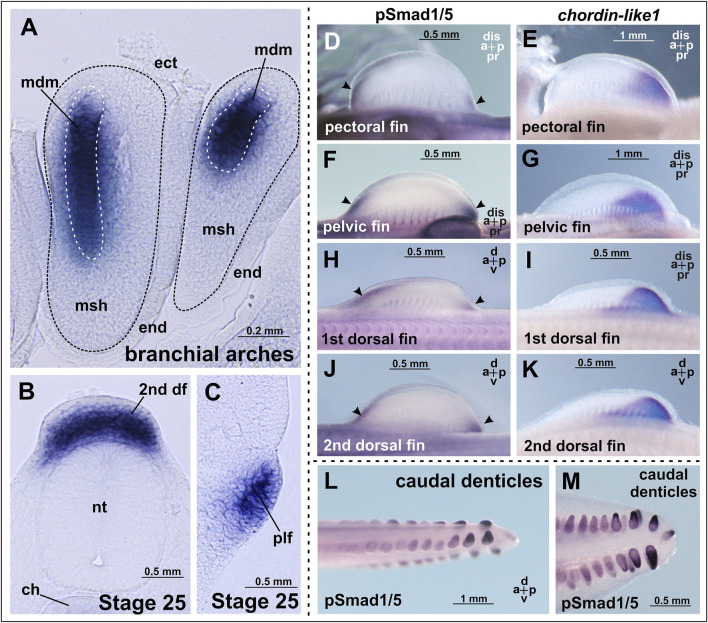
*Chordin-like1* expression in *C*. *griseum* embryos in the primordia of the branchial arches **(A)**, dorsal **(B)**, and pelvic **(C)** fins, shown on transverse sections. In the branchial arches, *chordin-like1* is expressed in the mesodermal core of the primordia. **(D–K)** Comparison of BMP signaling activity (assessed via phosphorylated Smad1/5 immunolabeling, arrowheads) and *chordin-like1* expression domains in the fin primordia of *C. griseum* at stages 28–29. In both paired and unpaired fins, BMP activity is observed in the distal anterior and posterior regions of the primordia, whereas *chordin-like1* expression is detected only in the posterior region, **(L, M)** BMP signaling activity in caudal denticles. The photo of the pelvic fin in panel **(G)** is the same as that in Figure 3X, and the photo of 2nd dorsal fin in panel **(K)** is the same as that in Figure 3Q. a–anterior, df–dorsal fin, dis–distal, ch–chord, ect–ectoderm, end–endoderm, msh–mesenchyme, mdm–mesoderm, nt–neural tube, p–posterior, plf–pelvic fin, pr–proximal.

At the earliest analyzed stage (stage 24), *chordin-like1* expression is observed in the branchial arches, including the mandibular and hyoid arches (Figure 3; Supplementary Figure S3). The expression is localized to the central region of the arches, which is derived from mesodermal tissue (Figure 4A).

By stage 25, expression is also detected in the primordia of the unpaired dorsal fins and, to a much weaker extent, in the paired pelvic fin buds (Figures 3, 4B,C). Notably, at this stage, the fins are not yet externally protruding structures.

At stage 26, when the dorsal fins begin to morphologically protrude, *chordin-like1* expression becomes localized to their caudal region. Expression is also seen in the caudal part of the pectoral fins and persists in the pelvic fins. Expression levels in the paired fins remain lower than in the gill arches and dorsal fins.

By stage 27, the paired fins become well-developed protrusions and *chordin-like1* expression within them increases (Figure 3). The expression pattern in paired and dorsal fins is similar, being localized to the caudal part of the fins. A low level of expression is also detected at the base of the anal fin and in the ventricle of the hindbrain.

A comparable pattern is observed at stage 28 (Figure 4). At this point, the paired and dorsal fins are morphologically similar, and *chordin-like1* expression is likewise similar in both. Expression in the branchial arches and fins persists at least until stage 31, the latest stage analyzed (Supplementary Figures S3E–H).

The presence of cis-rich domains in Chordin-like1 suggests its potential to function, similarly to Chordin, as an antagonist of the BMP signaling pathway. As modulation of BMP signaling has been described as an important factor in the development of both fins and limbs (Pizette et al., 2001; Mateus et al., 2020), we investigated the spatial relationship between regions of BMP activity and *chordin-like1* expression in shark fins to assess a potential role for Chordin-like1 in regulating BMP signaling. The pattern of BMP pathway activity in developing shark fins was assessed by detecting phosphorylated Smad1/5 (pSmad1/5), the intracellular mediators of BMP signaling, through immunohistochemical analysis. Phosphorylated Smad1/5 was detected in anterior regions of the fin that lacked *chordin-like1* expression, and in posterior margin, partially overlapping with *chordin-like1* (Figures 4D–K). The pSmad staining observed in our experiments in caudal denticles (Figures 4L,M) is consistent with published data on BMP pathway activity, and specifically with *bmp4* expression in the placode mesenchyme and dermal papilla of developing caudal denticles in sharks (Cooper et al., 2017; 2023).

### Expression of *chordin-like1* in sterlet

Another evolutionarily ancient lineage of jawed vertebrates is the Chondrostei, represented by the sterlet (*A*. *ruthenus*). The expression of *chordin-like1* was analyzed in embryos and prelarvae of sterlet by ISH.

**FIGURE 5:**
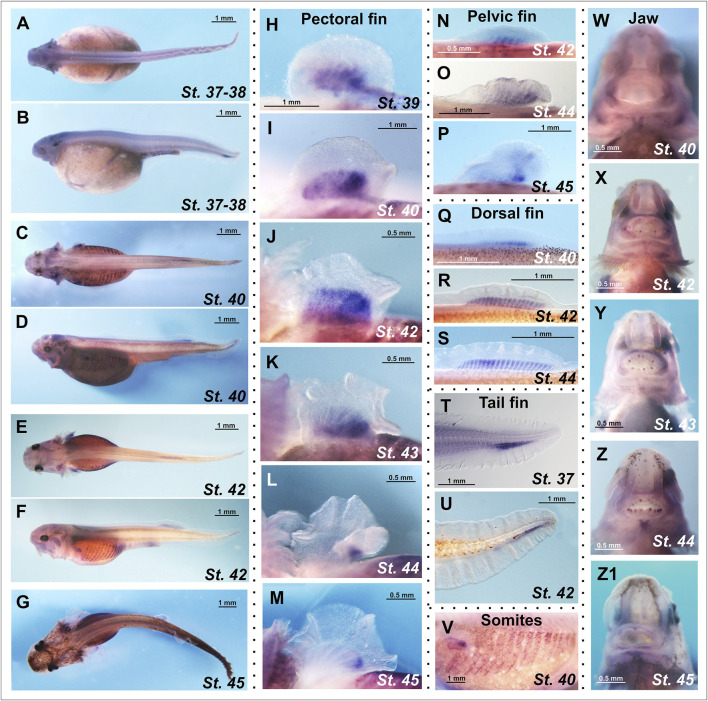
Spatial expression analysis of the *chordin-like1* gene in prelarval stages of the sterlet *Acipenser ruthenus*. **(A–G)** *Chordin-like1* expression in whole-mount prelarvae. **(H–M)** Expression of *chordin-like1* in the pectoral fins is observed in a broad domain corresponding to the formation of endoskeletal elements and displays a gradient pattern with a maximum in the posterior region of the fin **(H–K)**. As fin development progresses, *chordin-like1* expression becomes restricted to the posterior region, spatially corresponding to the location of the future metapterygial element **(L,M)**. **(N–P)** Expression of *chordin-like1* in the pelvic fins is observed in the domain of endoskeletal elements formation, with a maximum in the posterior region. **(Q–S)** Expression of *chordin-like1* in the dorsal fin is detected uniformly throughout the region of endoskeletal elements development. **(T, U)** Expression of *chordin-like1* in the caudal fin is observed in the region of the hypuralia. **(V)** Expression of chordin-like1 is detected in somites. **(W–Z1)** Expression of chordin-like1 is detected in jaw structures.

*Chordin-like1* expression in sterlet was detected in both paired and unpaired fins, specifically in the proximal regions corresponding to the presumptive domains of endoskeletal element formation (Figure 5). In the pectoral fins, *chordin-like1* expression at stages 39–42 displays a posteriorly biased gradient, with the highest expression in the caudal portion of the fin. At later stages (44–45), expression persists only in the posterior region, corresponding to the location of the metapterygial basal element (Figures 5H–M). A similar spatial pattern and temporal dynamic of expression were observed in the pelvic fins (Figures 5N–P).

In the dorsal fin, *chordin-like1* expression is localized to the endoskeletal region, specifically within the developing radials (Figures 5Q–S). In the heterocercal caudal fin, expression is observed in the area corresponding to the hypuralia (Figures 5T,U). Additionally, *chordin-like1* expression was detected in somites and jaw structures (Figures 5V–Z1).

### Expression of *chordin-like1* in clawed frog

Our data revealed that *chordin-like1* is most prominently expressed in the posterior region of paired fin buds in both shark and sterlet embryos. Remarkably, these regions in paired fins correspond to the presumptive location of the metapterygial basal elements, which are thought to have played a key role in the evolution of tetrapod limbs (Cass et al., 2021). In light of this, it is of particular interest to examine the expression pattern of *chordin-like1* orthologs in terrestrial vertebrates. We investigated this pattern during limb development in the African clawed frog *Xenopus laevis*, as representative of one of basal tetrapod lineage.

**FIGURE 6:**
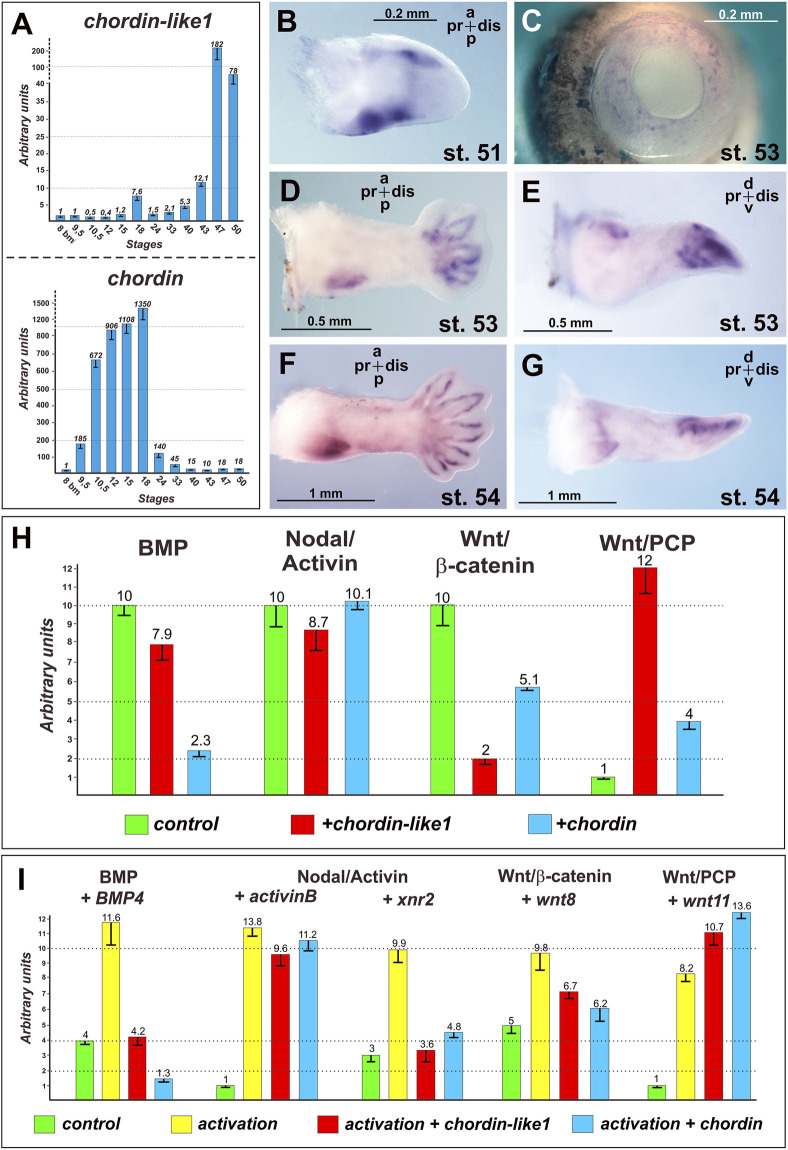
Expression and functional analysis of the Chordin-like1 in *Xenopus laevis*. Stages of *Xenopus laevis* are indicated according to Nieuwkoop and Faber (1994). **(A)** Analysis of *chordin-like1* expression dynamics in *Xenopus laevis* in comparison to *chordin* expression. A significant increase in *chordin-like1* expression is observed in tadpoles at stages 43–47, while the maximum expression of *chordin* occurs at significantly earlier stages: 10.5–18. **(B–G)** The spatial expression pattern of *chordin-like1* in the developing *Xenopus laevis* limb is first observed at stage 51 **(B)** in the marginal regions of the early limb bud. At later stages (53–54), expression becomes localized to the proximal-posterior region and the marginal zones of the digit primordia **(D–G)**. Additionally, chordin-like1 is expressed in the developing eye structures **(C)**. **(H)** Effects of *chordin-like1* mRNA injection on the activity of reporters for endogenous intracellular signaling pathways in luciferase assay compared with *chordin* mRNA effects. Compared to Chordin, Chordin-like1 exhibits a weaker inhibitory effect on the endogenous BMP pathway but a stronger inhibitory effect on the canonical Wnt/β-catenin pathway. Additionally, Chordin-like1 markedly activates the Wnt/PCP pathway. **(I)** Modulation of reporter activity for intracellular signaling cascades by *chordin-like1* in the context of their experimental activation by ligand co-injection in luciferase assay.

The temporal expression profile of *chordin-like1* in *X. laevis* was analyzed via RT-PCR and compared to that of the canonical *chordin* gene. As shown in Figure 6A, *chordin* is strongly upregulated during gastrulation and subsequently downregulated after the neurula stage. In contrast, *chordin-like1* expression is only markedly activated in tadpoles starting from stage 43. The early peak of *chordin* expression likely reflects its role as a major organizer of body axis formation, whereas the delayed activation of *chordin-like1* suggests its involvement in organogenesis.

The spatial expression pattern of *chordin-like1* was further investigated via ISH in *X. laevis* tadpoles. At stage 51, expression is detected along the anterior and posterior margins of the developing hindlimb bud, with stronger expression along the anterior and posterior edges (Figure 6B). By stage 53, *chordin-like1* is also expressed around the periphery of the emerging digital rays, forming a glove-like pattern (Figures 6D,E). Additionally, expression continues along the posterior-proximal margin of the limb bud, with a similar pattern persisting at stage 54 (Figures 6F,G). Beyond the limbs, *chordin-like1* expression is also observed in the developing eye fields (Figure 6C).

### Functional analysis of *Xenopus laevis* Chordin-like1 protein

To evaluate the functional activity of amphibian Chordin-like1, we assessed whether its mRNA is capable to induce secondary body axes in *X. laevis* embryos, a classical assay that has previously demonstrated the axis-inducing activity of Chordin and its orthologs (Schmidt et al., 1995; Piccolo et al., 1997).

Synthetic *chordin-like1* mRNA was injected into developing *X. laevis* embryos at the 8-cell stage. While *chordin-like1* mRNA injections resulted in phenotypic abnormalities, we did not observe the formation of secondary body axes (not shown), indicating that *chordin-like1* may not share the full axis-inducing potential of *chordin*.

To assess the capacity of *chordin-like1* to modulate the activity of BMP, Nodal/Activin, and Wnt signaling pathways, we performed luciferase reporter assays using a previously established protocol (Bairamov et al., 2011). Our data demonstrated that *chordin-like1* is capable of inhibiting the endogenous activity of the BMP signaling pathway, consistent with its ability to bind BMP4 molecules (Figure 6H; Supplementary Figure S4). In addition, *chordin-like1* modestly reduced the activity of the endogenous Nodal/Activin pathway. Remarkably, *chordin-like1* significantly suppressed the endogenous canonical Wnt/β-catenin pathway (by a factor of five), while robustly activating the non-canonical Wnt/PCP pathway (by a factor of ten) (Figure 6H).

Overall, under the experimental conditions applied, the effects of *chordin-like1* on the signaling pathways tested were generally similar to those of *Chordin*, though the magnitude of its influence varied between pathways. *Chordin-like1* exhibited a weaker effect on BMP pathway activity compared to *Chordin*, but demonstrated a more pronounced influence on both canonical and non-canonical Wnt signaling. Comparable results were observed upon experimental activation of the pathways using ligand mRNAs co-injected into *X. laevis* embryos along with the reporter constructs (Figure 6I).

### Loss of *chordin-like1* in Teleostei genomes correlates with reduction of metapterygium in their fins

Our analyses of phylogeny and local genomic synteny revealed the absence of *chordin-like1* in the majority of teleost fish, including the widely used model *Danio rerio*. The previously described *chordin-like* gene in *Danio* corresponds to *chordin-like2*, as confirmed by our data and consistent with earlier reports (Figure 2; Branam et al., 2010).

**FIGURE 7:**
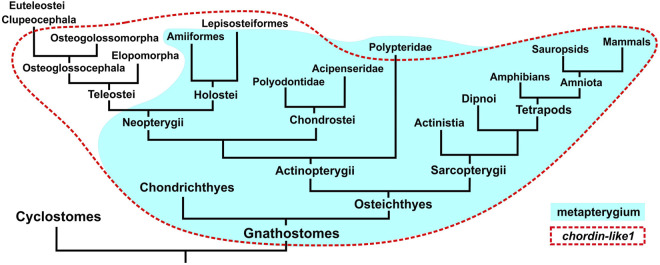
Comparison of gnathostome groups possessing a metapterygial element (or its derivatives) in the fins/limbs and the presence of the *chordin-like1* gene in their genomes.

Given the expression patterns observed in cartilaginous and sturgeon species, where *chordin-like1* may be involved in development of metapterygial element, we propose that the evolutionary loss of *chordin-like1* in teleosts can be associated with the reduction of this structure during the fin evolutionary specialization process (Hawkins et al., 2021). In a schematic phylogenetic tree of jawed vertebrates, the presence of *chordin-like1* and the metapterygium largely overlap (Figure 7). Notable exceptions include Polypteridae, which retain the metapterygium despite the loss of *chordin-like1*, and conversely, gars and basal teleost groups such as Elopomorpha and Osteoglossomorpha, which have reduced metapterygium but retain *chordin-like1*. In most euteleost fishes, the loss of *chordin-like1* correlates with metapterygial reduction.

## Discussion

### *Chordin-like1* is a gnathostome-specific gene expressed in evolutionarily novel morphological structures in basally divergent representatives of the clade

As we demonstrated through phylogenetic and local genomic synteny analyses, *chordin-like1* is a gnathostome-specific gene that likely emerged as a result of duplication of the evolutionarily more ancient *chordin-like2*. Since the body plan of gnathostomes is characterized by a number of key morphological innovations, including paired appendages and a jaw apparatus, we hypothesize that the emergence of *chordin-like1*, alongside other genomic changes, may have contributed to the origin and evolution of these structures. This hypothesis is supported by previously published data showing *chordin-like1* expression in the limb buds of chick and mouse embryos (Allen et al., 2013; Coffinier et al., 2001; Nakayama et al., 2001).

To further test the potential role of *chordin-like1* in the evolution of paired appendages and jaws, we analyzed the expression of *chordin-like1* orthologs in representatives of extant basally divergent gnathostome groups - cartilaginous fishes and chondrostean. These taxa are particularly valuable for the study of vertebrate appendage evolution, as the tribasal pectoral fins of cartilaginous fishes, and the structurally similar fins of chondrostean, are considered a basal model of vertebrate paired fins based on their endoskeletal organization (Cole and Currie, 2007; Davis et al., 2004). It is from this ancestral fin type that the divergent fin structures of actinopterygians and the limbs of tetrapods are believed to have evolved, accompanied by the reduction of certain basal elements and the elaboration of others (Ahn and Ho, 2008; Zhang et al., 2012).

Our analysis revealed the following features of *chordin-like1* expression in the fins of the gray catshark and the sterlet:

1. In shark embryos, *chordin-like1* expression exhibits a distinctly localized pattern, being restricted to only a few embryonic structures - the branchial arches and fin buds - suggesting a possible similarity in the mechanisms of formation of these structures. At later stages, additional expression is observed in the hindbrain ventricle.
2. Expression of *chordin-like1* in the dorsal fin buds begins earlier than in the paired fin buds, but the expression pattern in both cases is highly similar, being localized to the posterior part of the fin bud. These findings are consistent with the hypothesis that paired fins co-opted portions of the developmental program of median fins, which are thought to have evolved earlier (Cass et al., 2021; Abe and Ota, 2017).
3. In the sterlet, *chordin-like1* is expressed at comparatively late stages of fin buds development, when endoskeletal elements begin to form and differentiate. Similar to the shark, *chordin-like1* is expressed in the paired (pectoral and pelvic) and median (dorsal) fins of the sterlet.

In the sterlet pectoral fins, *chordin-like1* expression initially occupies a broader domain and extends more anteriorly compared to shark fins. During early stages, the expression exhibits a gradient-like pattern with the highest levels in the posterior region. As development proceeds, *chordin-like1* expression in the sterlet pectoral fins becomes progressively restricted to the posterior region of the fin, corresponding to the developing metapterygial element. Studies of cartilage development in the pectoral fins of sturgeons have shown that the elements of the future metapterygium form first, followed by the appearance of a surrounding chondrogenic mesenchymal disc, characteristic of actinopterygian fins. Thus, in terms of developmental mechanisms, sturgeon fins represent a composite structure incorporating basal elements of both actinopterygian and sarcopterygian fins (Davis et al., 2004).

In sterlet pelvic fins, *chordin-like1* expression shows a pattern similar to that in the pectoral fins, though the pelvic fins are smaller and develop later in ontogeny (Zhang et al., 2012). The observed expression of *chordin-like1* in unpaired fins of the starlet, specifically in the radial elements of the dorsal fin and the hypurals of the caudal fin, may also support the hypothesis of shared developmental mechanisms between paired and unpaired fins (Freitas et al., 2006; Hawkins et al., 2022).

In addition to fin structures, in the shark we observed *chordin-like1* expression in the central mesodermal core (as seen in cross-sections) of the branchial arch primordia (Figure 5A). However, we did not detect similar expression in the branchial arches of the sterlet. This difference may be attributed to the fact that, in sturgeons, the mesodermal core of the developing branchial arches is either poorly defined or absent, and the central part of the arch is mainly formed from mesenchymal derivatives of neural crest cells (Gillis et al., 2013).

### Chordin-like1 is involved in limb development in *Xenopus laevis*

In the paired fin buds of catshark and sterlet embryos, *chordin-like1* was expressed in the presumptive metapterygial basal element, which is believed to be the evolutionary precursor of the tetrapod limb. This observation prompted us to investigate *chordin-like1* expression during limb development in a representative of a basal tetrapod lineage—the African clawed frog (*X*. *laevis*).

Our analysis revealed that *chordin-like1* is expressed at the limb margin during the early stages of limb bud formation and remains active at the distal margin during digit morphogenesis. It is well established that mutual regulation of BMP, Wnt, and Sox9 signaling plays a critical role in both digit formation in tetrapod limbs and in the development of radial elements in shark fins (Raspopovic et al., 2014; Onimaru et al., 2016; Hiscock et al., 2017). BMP and Wnt activities are localized to the interdigital regions (Satoh et al., 2005), while *Sox9* expression marks the chondrogenic mesenchyme and the formation of skeletal elements (Montero et al., 2017).

Our luciferase reporter assays demonstrated that Chordin-like1 can modulate endogenous BMP and Wnt signaling pathways, suggesting its possible involvement in the morphogenesis of limb structures, including the autopod and digits. Furthermore, partially complementary spatial patterns of *chordin-like1* expression and BMP signaling activity observed in shark fin buds via immunohistochemical analysis support the hypothesis that *chordin-like1* may act as a BMP antagonist during fin development.

In our axis duplication assays, *X. laevis chordin-like1* mRNA did not induce secondary axes in *Xenopus* embryos. Structurally, *сhordin-like1* in *X. laevis* contains two cysteine-rich (CR) domains involved in BMP binding, whereas most *chordin-like1* orthologs in other species contain three CR domains (Supplementary Figure S1; Larraín et al., 2000). While murine *Chordin-like1* has been shown to induce secondary axes in *Xenopus* (Coffinier et al., 2001; Nakayama et al., 2001), the lack of such activity in the *Xenopus сhordin-like1* ortholog may be attributed to its structural and functional divergence.

Nevertheless, our luciferase assays revealed that Chordin-like1 is capable of suppressing BMP signaling activity, albeit much less potently than *Chordin*, and can bind BMP4 ligands (Figures 6L,M; Supplementary Figure S2), consistent with previous reports (Nakayama et al., 2001). Its minimal effect on Activin activity also aligns with data indicating that Chordin-like1 does not bind Activin (Nakayama et al., 2001).

Interestingly, Chordin-like1 exhibited the strongest effects on Wnt signaling: it inhibited the canonical pathway while activating the non-canonical branch. The involvement of Wnt signaling in limb bud development and limb mesenchyme patterning has been previously reported (Lovely et al., 2022; Glotzer et al., 2022; Geetha-Loganathan et al., 2008; ten Berge et al., 2008). The exact mechanism by which Chordin-like1 modulates Wnt signaling in this context remains unclear and warrants further investigation in future studies.

The expression of *chordin-like1* in the developing eye observed in our study is consistent with previous findings in both mouse and *Xenopus* (Sakuta et al., 2001; Pfirrmann et al., 2015). Moreover, mutations in the human *CHRDL1* gene have been associated with X-linked megalocornea (MGC1) disorder (Davidson et al., 2014; Webb et al., 2012; Han et al., 2015).

### Chordin-like1 may be involved in the development of metapterygial limb derivatives

Our phylogenetic analysis revealed a correlation between the loss of the *chordin-like1* gene in teleost fishes and the reduction of the metapterygium in their fins. It has been shown that the pectoral fins of teleosts undergo indirect development, involving metamorphosis, such that the structure of the embryonic and adult fins differs (Grandel and Schulte-Merker, 1998). At the larval stage, the endoskeletal elements of the fin are represented by an undifferentiated endoskeletal disc, while the adult fin develops later, post-metamorphosis, although exceptions to this dominant pattern have been reported (Shkil et al., 2022). A similar biphasic developmental pattern and lack of direct continuity between the larval fin fold and the adult fin has been described for the dorsal fin in teleosts (Miyamoto et al., 2022). These findings suggest that the molecular mechanisms underlying fin development in teleosts may possess unique features that deviate from the basal pattern of vertebrate limb development, which may account for the absence of *chordin-like1* in this clade. The absence of this gene in teleosts is likely a secondary loss, as it is retained in the genomes of more basal fish lineages, including basal actinopterygians such as gars. *Chordin-like1* is also preserved in some of the most evolutionarily ancient teleosts, including representatives of Elopomorpha and Osteoglossomorpha.

Additional evidence that genes of the *Chordin* family might be associated with the emergence of paired appendages in vertebrates comes from studies on the goldfish (*Carassius auratus*), where a mutation in one of the *Chordin* paralogs (*Chordin* is duplicated in Teleostei in result of the teleost-specific WGD round) leads to the duplication of caudal and anal fins due to disrupted translation of the full-length protein (Abe et al., 2014). This mutation, in its homozygous state, became fixed through long-term (approximately 600 years) artificial selection under domestication in China. These findings may point to a continuity in developmental mechanisms between the evolutionarily older median fins and the more derived paired fins.

According to our data, *chordin-like1* is not a gene exclusively specific to paired fins, as it is also expressed in unpaired fins and in the primordia of the gill arches and the mandibular arch, which is derived from the anterior gill arches (Gillis et al., 2013). This expression pattern of *chordin-like1* may reflect a similarity in the fundamental developmental mechanisms of these structures in gnathostomes. Unlike paired fins, unpaired fins are present in jawless vertebrates and are therefore considered evolutionarily more ancient (Freitas et al., 2014). At the same time, the homology between unpaired fins of jawless vertebrates and those of gnathostomes remains a subject of debate (Kariyayama et al., 2024). In both lampreys and gnathostomes, the development of unpaired fins is based on the paraxial (somitic) mesoderm (Freitas et al., 2006; Shimada et al., 2013). However, in contrast to the fins of lampreys, gnathostome fins are massive structures that have evolved into the limbs of terrestrial vertebrates, possessing a full three-dimensional structure differentiated along the proximodistal, anteroposterior, and dorsoventral axes (McQueen and Towers, 2020; Castilla-Ibeas et al., 2024; Cooper et al., 2023; Zeller et al., 2009; Sheeba et al., 2016). Thus, the evolutionary trajectory of vertebrate limbs can be viewed as a transition from a fin fold - a flat, two-dimensional fin that passively stabilizes and increases body surface area in an aquatic environment - to a complex, three-dimensionally structured limb adapted for active locomotion in space. The structure of gnathostome appendages is heterogeneous. During evolution, basal elements were reduced independently and differently in the lineages of teleosts and tetrapods. In teleosts, the posteriorly positioned metapterygium was lost, whereas in tetrapods, by contrast, the anterior propterygium and mesopterygium were reduced, while only the metapterygium was retained and further developed. This observation may indicate a certain degree of autonomy in the regulatory modules governing the development of anterior and posterior elements of fins. The emergence of structural heterogeneity, first in unpaired fins and subsequently in paired fins (following their sequential appearance in the fossil record), could have involved novel regulatory factors such as *chordin-like1*. Based on the observed expression pattern of *chordin-like1* in the mesodermal core of gill arch primordia of sharks, this gene may be associated with the development of mesodermal tissue, which forms the basis of endoskeletal fin elements. In the posterior region of both paired and unpaired fins, there is a signaling center known as the zone of polarizing activity (ZPA), which serves as a source of Shh signaling involved in mesoderm development (Letelier et al., 2021; Hawkins et al., 2022). It can be hypothesized that the *chordin-like1*, which first emerged in gnathostomes, may have contributed to the formation of a regulatory network that initially enabled the development of massive unpaired fins and, later - through co-option of this established regulatory network from the paraxial mesoderm (of unpaired fins) into the lateral plate mesoderm - facilitated the emergence and further evolutionary transformation of paired fins.

An alternative scenario might suggest that the activity of *chordin-like1* is not part of the core developmental mechanism of gnathostome appendages, but rather a secondary feature that emerged in the fin development of certain lineages. However, in our view, the presence of shared features in the expression patterns of *chordin-like1* in representatives of two basally divergent gnathostome groups (Chondrichthyes and Chondrostei), together with evidence of this gene’s expression in the limbs of more evolutionarily derived lineages, may indicate that *chordin-like1* became integrated into the regulatory network of appendage development from the early stages of gnathostome evolution.

## Materials and methods

### Animals and samples preparation

The animal study protocol was approved by the Institutional Review Board (or Ethics Committee) of the Shemyakin-Ovchinnikov Institute of Bioorganic Chemistry (Moscow, Russia, protocol code IACUC 229 dated 1 February 2018). The study was conducted in accordance with the local legislation and institutional requirements.

*C. griseum* eggs and embryos were collected in collaboration with the scientific department of the Moskvarium Center for Oceanography and Marine Biology (Moscow, Russia). The embryos of *C. griseum* were staged in accordance with Ballard et al., 1993.

The *A. ruthenus* eggs and embryos were obtained and collected in Tver district, Konakovo, Russia. The embryos of *A. ruthenus* were staged in accordance with in accordance with Ginzburg and Detlaf (1975) and Shmalgauzen (1975).

*X. laevis* embryos are kept in the IBCH vivarium on a permanent basis. *X. laevis* were staged after Nieuwkoop and Faber, 1994.

For ISH, embryos were fixed in MEMFA solution (3.7% formaldehyde, 100 mM MOPS, 2 mM EGTA, 1 mM MgSO4), dehydrated in methanol and kept at −20°C.

*X. laevis* total RNA samples of set of stages were obtained from lysed embryos (10 embryos for probe) by purification with the Analytic Jena innuPREP RNA Mini Kit 2.0 (Berlin, Germany).

### Analyses of phylogeny and local genomic synteny

The search for homologs was carried out in Blastn (https://blast.ncbi.nlm.nih.gov/Blast.cgi?PROGRAM=blastn&PAGE_TYPE=BlastSearch&BLAST_SPEC=&LINK_LOC=blasttab&LAST_PAGE=blastn) and tBlastn (https://blast.ncbi.nlm.nih.gov/Blast.cgi?PROGRAM = tblastn&PAGE_TYPE = BlastSearch&BLAST_SPEC = &LINK_LOC = blasttab&LAST_PAGE = blastn) sections. We checked available Nucleotide collections (nr/nt) and whole genome shotgun contigs (wgs).

Multiple alignment was performed by ClustalW algorhythm in the MEGA11 program.

Phylogenetic analyses of Chordin-like1 protein sequences of vertebrates were performed via the Maximum Likehood (ML) methods using the MEGA11 program (Tamura et al., 2021).

The choosing of optimal model was made in MEGA11.

In ML method JTT matrix-based model (Jones et al., 1992) with frequencies and Gamma distribution was used. The percentage of trees in which the associated taxa clustered together in the bootstrap test (500 replicates) is shown next to the branches (Felsenstein, 1985). The tree is drawn to scale, with branch lengths measured in the number of substitutions per site. This analysis involved 60 amino acid sequences.

The list of the analyzed Chordin-like1 sequences is attached in Supplementary Info.

Synteny analysis and search for neighboring genes were also carried out on the NCBI website (https://www.ncbi.nlm.nih.gov/).

### *Chordin-like1* cDNA obtaining, RT-PCR, ISH

*Chiloscyllium griseum Chordin-like1* cDNA was obtained by PCR with following primers:

Cg_ChL1_full_Frw1; ATTGAATTCGCCACCATGAGAGCGAGCTGGAGACTG; 0.04.

Cg_ ChL1_full_Rev1; AATGTCGACTCAGCAGTGCTCCTTTTCTGG; 0.04.

*Acipenser ruthenus Chordin-like1* cDNA was obtained by PCR with following primers:

AR_ChL1_full_Frw2; ATTGAATTCGCCACCATGACAGGGGTGTGCAGACT; 0.04.

AR_ ChL1_full_Rev2; AATCTCGAGTCAGCAGTGCCCTTTCATTG; 0.04.

*Xenopus laevis Chordin-like1* cDNA was obtained by PCR with following primers:

Xl_ChL1_full_Frw1; ATTAGATCTGCCACCATGTTGGACTTGGCCAGTG; 0.04.

Xl_ ChL1_full_Rev1; AATCTCGAGTTAACAATGGCCCTTTTCAC; 0.04.

PCR was performed with Encyclo polymerase Evrogen kit (www.evrogen.ru, Moscow).

The resulting cDNA fragments were cloned into the pAL2-T vector (Evrogen, Moscow) and cDNA inserts of 3 clones of each gene were sequenced.

To obtain mRNA for injection *X. laevis chordin-like1* cDNAs was recloned into the pCS2 vector. mRNA synthesis was carried out by SP6 mMessage mMachine kit (Thermo Fisher Scientific, Waltham, Massachusetts).

ISH was carried out according to the protocol described by Bayramov et al. (2011), Ermakova et al. (2020) and Ermakova et al. (2024).

For the analysis of *chordin-like1* expression patterns in ISH, at least 5 *C. griseum* embryos and at least 10 *A. rutenus* prelarvae or *X. laevis* tadpoles from each of the presented stages were analysed. Expression patterns that were reproduced in at least 80% of cases were considered reliable.

Photography was carried out using a Leica M205 stereo microscope.

For qRT-PCR analysis first strand samples were obtained using total RNA samples collected in three repetitions. The concentration of the extracted RNA was measured with a Qubit® fluorometer (Invitrogen), while RNA integrity was checked visually via gel electrophoresis.

Three independent pairs of primers were used for *X. laevis chordin-like1* to exclude unspecific signals. The following pairs of primers designed by Primer-Blast tool on the base of *X. laevis chordin-like1* sequence were used:

Xl_ChL1_RT1_Frw; GGAGTGGTGGAATGTGTGCT; 0.04.

Xl_ChL1_ RT1_Rev; GTCCCCAGAATGAGGGTCAA; 0.04.

Xl_ChL1_ RT2_Frw; GACGACACCAAGTCAGTGGA; 0.04.

Xl_ChL1_ RT2_Rev; TGGCCCTTTTCACTCTTGTCT; 0.04.

Xl_ChL1_ RT3_Frw; GGCATTCTGCGCCAGTTTTA; 0.04.

Xl_ChL1_ RT3_Rev; GCGGCTTATTTGTGATTCCCC; 0.04.

Each qRT-PCR test was performed in triplicate.

### Luciferase assay, co-immunoprecipitation, and immunohistochemical analysis

The luciferase assay and co-immunoprecipitation were performed as previously described (Bayramov et al., 2011; Eroshkin et al., 2016). Briefly, for the luciferase assay, *X. laevis* embryos at the two-to four-cell stage were injected with a mixture containing one of the luciferase reporter plasmids [GL3-ARE-Luc (Pierreux et al., 2000); TOPflash, Millipore; or TCFm-Luc (Hikasa et al., 2010)], the reference plasmid pCMV-β-GAL (50 pg per embryo of each plasmid), and the corresponding mRNA.

Nodal/Activin signaling was activated by microinjection of mRNA encoding Activin and/or Xnr2, known endogenous inducers of this pathway. Wnt signaling was activated via injection of Wnt8 or Wnt11 mRNA.

Following injection, *X. laevis* embryos were incubated for approximately 16 h at 14°C until reaching mid-to late-gastrula stages (stage 11–12 according to Nieuwkoop and Faber, 1994) in 0.1× MMR solution (Mark’s Modified Ringer’s, as described in Sive et al., 2000).

Each experimental condition for luciferase assay was analyzed in triplicate, with 10 embryos per replicate.

For lysate preparation, embryos were homogenized on ice in Cell Culture Lysis Reagent (Promega) consisting of 25 mM Tris-phosphate (pH 7.8), 2 mM DTT, 2 mM 1,2-diaminocyclohexane-N,N,N′,N′-tetraacetic acid, 10% glycerol, and 1% Triton® X-100. Homogenization was performed at a volume of 10 µL per embryo or 5 µL per explant, followed by centrifugation at 13,400 rpm for 30 min at 4°C.

Luciferase activity was measured using the Luciferase Assay System (Promega) according to the manufacturer’s protocol. Typically, 5–8 µL of lysate and 25 µL of stabilized luciferin substrate were used per reaction. Luminescence was detected using a TD-20/20 Luminometer (Turner Designs).

For co-immunoprecipitation, Flag- and Myc-tagged proteins were expressed by injecting *X. laevis* embryos with synthetic mRNAs encoding the respective tagged constructs, with cloning strategies as described in Bayramov et al. (2011).

Immunohistochemistry on *C*. *griseum* was performed as previously reported (Blackiston et al., 2010; Orlov et al., 2022), with extended washing steps (five washes for 1 h each at room temperature). NBT/BCIP was used as a chromogenic substrate. The primary antibody (anti-pSMAD1/5/8, Cell Signaling) was used at a 1:200 dilution, and the secondary antibody (alkaline phosphatase-conjugated anti-rabbit IgG, Sigma) at a 1:2000 dilution.

## Data Availability

The original contributions presented in the study are included in the Article/Supplementary Material, further inquiries can be directed to the corresponding author.

## Supplementary material

SUPPLEMENTARY FIGURE S1 Schematic representation of the arrangement of conserved cis-rich domains (CR, https://www.ncbi.nlm.nih.gov/Structure/cdd/cddsrv.cgi?uid=214564; Garcia Abreu et al., 2002) and chordin (CHRD, 2003) domains in Chordin (Chrd) and Chordin-like (Ch-L) proteins across vertebrates, cephalochordates, and echinoderms.

SUPPLEMENTARY FIGURE S2 Analysis of local genomic synteny of *chordin-like* genes in Chondrichthyes and Teleostei. 
**Chondrichthyes species**, *Carcharodon carcharias*, *Callorhinchus milii*, *Chiloscyllium griseum*, *Chiloscyllium punctatum*, *Chiloscyllium plagiosum*, *Hemitrygon akajei*, *Hemiscyllium ocellatum*, *Heptranchias perlo*, *Leucoraja erinacea*, *Mobula birostris*, *Pristiophorus japonicas*, *Pristis pectinata*, *Rhincodon typus*, *Scyliorhinus canicula*, *Scyliorhinus torazame*, *Stegostoma tigrinum*.
**Teleostei species**, *Anguilla anguilla*, *Antennarius striatus*, *Chanos chanos*, *Clarias gariepinus*, *Danio rerio*, *Denticeps clupeoides*, *Esox lucius*, *Gadus morhua*, *Megalops cyprinoides*, *Perca flavescens*, *Scleropages formosus*, *Takifugu rubripes*.

SUPPLEMENTARY FIGURE S3
*Chordin-like1* expression in *C*. *griseum* embryos in branchial arches at stages 24-27 (A-D) and in fins and branchial arches at stage 31 (E-H). a – anterior, ba – branchial arches, hy – hyoid arch, md – mandibular arch, df – dorsal fin, pcf – pectoral fin, plf – pelvic fin, p – posterior.

SUPPLEMENTARY FIGURE S4 Chordin-like1 (Ch-L1), similar to Chordin, is able to bind BMP4. Myc-tagged Chordin-like1 protein (Ch-L1) binds flag-tagged BMP4 in CoIP assay (left panel, main signal is about 50-55 kDa), similar to Chordin (right panel).

## References

[B1] Abe G. Ota K. G. (2017). Evolutionary developmental transition from median to paired morphology of vertebrate fins: perspectives from twin-tail goldfish. Dev. Biol. 427 (2), 251–257. 10.1016/j.ydbio.2016.11.022 27939770

[B2] Abe G. Lee S. H. Chang M. Liu S. C. Tsai H. Y. Ota K. G. (2014). The origin of the bifurcated axial skeletal system in the twin-tail goldfish. Nat. Commun. 5, 3360. 10.1038/ncomms4360 24569511 PMC3948052

[B3] Ahn D. Ho R. K. (2008). Tri-phasic expression of posterior Hox genes during development of pectoral fins in zebrafish: implications for the evolution of vertebrate paired appendages. Dev. Biol. 322 (1), 220–233. 10.1016/j.ydbio.2008.06.032 18638469

[B4] Allen J. M. McGlinn E. Hill A. Warman M. L. (2013). Autopodial development is selectively impaired by misexpression of chordin-like 1 in the chick limb. Dev. Biol. 381 (1), 159–169. 10.1016/j.ydbio.2013.06.003 23764427

[B5] Balfour F. M. (1881). On the development of the skeleton of the paired fins of Elasmobranchii, considered in relation to its bearing on the nature of the limbs of the vertebrata. Proc. Zool. Soc. Lond. 1881, 656–671.

[B6] Ballard W. W. Mellinger J. Lechenault H. (1993). A series of normal stages for development of *Scyliorhinus canicula*, the lesser spotted dogfish (Chondrichthyes: scyliorhinidae). J. Exp. Zool. 267 (3), 318–336. 10.1002/jez.1402670309

[B7] Bayramov A. V. Eroshkin F. M. Martynova N. Y. Ermakova G. V. Solovieva E. A. Zaraisky A. G. (2011). Novel functions of Noggin proteins: inhibition of Activin/Nodal and Wnt signaling. Development 138 (24), 5345–5356. 10.1242/dev.068908 22071106

[B8] Bayramov A. V. Ermakova G. V. Eroshkin F. M. Kucheryavyy A. V. Martynova N. Y. Zaraisky A. G. (2016). The presence of Anf/Hesx1 homeobox gene in lampreys suggests that it could play an important role in emergence of telencephalon. Sci. Rep. 23 (6), 39849. 10.1038/srep39849 28008996 PMC5180219

[B9] Bayramov A. V. Yastrebov S. A. Mednikov D. N. Araslanova K. R. Ermakova G. V. Zaraisky A. G. (2024). Paired fins in vertebrate evolution and ontogeny. Evol. Dev. 26, e12478. 10.1111/ede.12478 38650470

[B10] Blackiston D. Vandenberg L. N. Levin M. (2010). High-throughput *Xenopus laevis* immunohistochemistry using agarose sections. Cold Spring Harb. Protoc. 2010 (12), prot5532. 10.1101/pdb.prot5532 21123419 PMC3654656

[B11] Boulet A. M. Moon A. M. Arenkiel B. R. Capecchi M. R. (2004). The roles of Fgf4 and Fgf8 in limb bud initiation and outgrowth. Dev. Biol. 273 (2), 361–372. 10.1016/j.ydbio.2004.06.012 15328019

[B12] Branam A. M. Hoffman G. G. Pelegri F. Greenspan D. S. (2010). Zebrafish chordin-like and chordin are functionally redundant in regulating patterning of the dorsoventral axis. Dev. Biol. 341 (2), 444–458. 10.1016/j.ydbio.2010.03.001 20226780 PMC2862114

[B13] Capdevila J. Tsukui T. Rodríquez Esteban C. Zappavigna V. Izpisúa Belmonte J. C. (1999). Control of vertebrate limb outgrowth by the proximal factor Meis2 and distal antagonism of BMPs by Gremlin. Mol. Cell 4 (5), 839–849. 10.1016/s1097-2765(00)80393-7 10619030

[B14] Casanova J. C. Uribe V. Badia-Careaga C. Giovinazzo G. Torres M. Sanz-Ezquerro J. J. (2011). Apical ectodermal ridge morphogenesis in limb development is controlled by Arid3b-mediated regulation of cell movements. Development 138 (6), 1195–1205. 10.1242/dev.057570 21307092

[B15] Cass A. N. Elias A. Fudala M. L. Knick B. D. Davis M. C. (2021). Conserved mechanisms, novel anatomies: the developmental basis of fin evolution and the origin of limbs. Diversity 13 (8), 384. 10.3390/d13080384

[B114] Castilla-Ibeas A. Zdral S. Oberg K. C. Ros M. A. (2024). The limb dorsoventral axis: Lmx1b’s role in development, pathology, evolution, and regeneration. Dev. Dynam. 253 (9), 798–814. 10.1002/dvdy.695 PMC11656695 38288855

[B16] Chrd H. M. (2003). CHRD, a novel domain in the BMP inhibitor chordin, is also found in microbial proteins. Trends Biochem. Sci. 28 (9), 470–473. 10.1016/S0968-0004(03)00171-3 13678956

[B17] Christen B. Rodrigues A. M. Monasterio M. B. Roig C. F. Izpisua Belmonte J. C. (2012). Transient downregulation of Bmp signalling induces extra limbs in vertebrates. Development 139 (14), 2557–2565. 10.1242/dev.078774 22675213

[B18] Coates M. I. (2003). The evolution of paired fins. Theory Biosci. 122, 266–287. 10.1007/s12064-003-0057-4

[B19] Coffinier C. Tran U. Larraín J. De Robertis E. M. (2001). Neuralin-1 is a novel Chordin-related molecule expressed in the mouse neural plate. Mech. Dev. 100 (1), 119–122. 10.1016/s0925-4773(00)00507-4 11118896

[B20] Cole N. J. Currie P. D. (2007). Insights from sharks: evolutionary and developmental models of fin development. Dev. Dyn. 236 (9), 2421–2431. 10.1002/dvdy.21268 17676641

[B21] Cooper R. L. Martin K. J. Rasch L. J. Fraser G. J. (2017). Developing an ancient epithelial appendage: FGF signalling regulates early tail denticle formation in sharks. EvoDevo 8, 8. 10.1186/s13227-017-0071-0 28469835 PMC5414203

[B22] Cooper R. L. Nicklin E. F. Rasch L. J. Fraser G. J. (2023). Teeth outside the mouth: the evolution and development of shark denticles. Evol. Dev. 25 (1), 54–72. 10.1111/ede.12427 36594351

[B23] Crossley P. H. Minowada G. MacArthur C. A. Martin G. R. (1996). Roles for FGF8 in the induction, initiation, and maintenance of chick limb development. Cell 84 (1), 127–136. 10.1016/s0092-8674(00)80999-x 8548816

[B24] Dahn R. D. Davis M. C. Pappano W. N. Shubin N. H. (2007). Sonic hedgehog function in chondrichthyan fins and the evolution of appendage patterning. Nature 445 (7125), 311–314. 10.1038/nature05436 17187056

[B25] Davidson A. E. Cheong S. S. Hysi P. G. Venturini C. Plagnol V. Ruddle J. B. (2014). Association of CHRDL1 mutations and variants with X-linked megalocornea, Neuhäuser syndrome and central corneal thickness. PLoS One 9 (8), e104163. 10.1371/journal.pone.0104163 25093588 PMC4122416

[B26] Davis M. C. Shubin N. H. Force A. (2004). Pectoral fin and girdle development in the basal actinopterygians *Polyodon spathula* and *Acipenser transmontanus* . J. Morphol. 262 (2), 608–628. 10.1002/jmor.10264 15376275

[B27] De Robertis E. M. Moriyama Y. (2016). The chordin morphogenetic pathway. Curr. Top. Dev. Biol. 116, 231–245. 10.1016/bs.ctdb.2015.10.003 26970622

[B28] De Robertis E. M. Moriyama Y. Colozza G. (2017). Generation of animal form by the chordin/tolloid/BMP gradient: 100 years after D’arcy Thompson. Dev. Growth Differ. 59 (7), 580–592. 10.1111/dgd.12388 28815565

[B29] Dealy C. N. Seghatoleslami M. R. Ferrari D. Kosher R. A. (1997). FGF-stimulated outgrowth and proliferation of limb mesoderm is dependent on syndecan-3. Dev. Biol. 184 (2), 343–350. 10.1006/dbio.1997.8525 9133440

[B30] Diogo R. (2020). Cranial or postcranial-Dual origin of the pectoral appendage of vertebrates combining the fin-fold and gill-arch theories? Dev. Dyn. 249 (10), 1182–1200. 10.1002/dvdy.192 32395826

[B31] DuBuc T. Q. Ryan J. F. Martindale M. Q. (2019). Dorsal-Ventral genes are part of an ancient axial patterning system: evidence from *Trichoplax adhaerens* (Placozoa). Mol. Biol. Evol. 36 (5), 966–973. 10.1093/molbev/msz025 30726986 PMC6501881

[B32] Enny A. Flaherty K. Mori S. Turner N. Nakamura T. (2020). Developmental constraints on fin diversity. Dev. Growth Differ. 62 (5), 311–325. 10.1111/dgd.12670 32396685 PMC7383993

[B33] Ermakova G. V. Kucheryavyy A. V. Zaraisky A. G. Bayramov A. V. (2020). Discovery of four Noggin genes in lampreys suggests two rounds of ancient genome duplication. Commun. Biol. 3 (1), 501. 10.1038/s42003-020-01234-3 32913324 PMC7483449

[B34] Ermakova G. V. Meyntser I. V. Zaraisky A. G. Bayramov A. V. (2024). Adaptation of the *in situ* hybridization method for working with embryos and larvae of modern representatives of phylogenetically ancient groups of vertebrates: lampreys, cartilaginous fishes and sturgeons. Russ. J. Dev. Biol. 55, 284–295. 10.1134/S1062360424700255

[B35] Eroshkin F. M. Nesterenko A. M. Borodulin A. V. Martynova N. Y. Ermakova G. V. Gyoeva F. K. (2016). Noggin4 is a long-range inhibitor of Wnt8 signalling that regulates head development in *Xenopus laevis* . Sci. Rep. 6, 23049. 10.1038/srep23049 26973133 PMC4789793

[B36] Feneck E. Logan M. (2020). The role of retinoic acid in establishing the early limb bud. Biomolecules 10 (2), 312. 10.3390/biom10020312 32079177 PMC7072211

[B37] Fernandez-Teran M. Ros M. A. (2008). The apical ectodermal ridge: morphological aspects and signaling pathways. Int. J. Dev. Biol. 52 (7), 857–871. 10.1387/ijdb.072416mf 18956316

[B38] Freitas R. Zhang G. Cohn M. J. (2006). Evidence that mechanisms of fin development evolved in the midline of early vertebrates. Nature 442 (7106), 1033–1037. 10.1038/nature04984 16878142

[B39] Freitas R. Gómez-Skarmeta J. L. Rodrigues P. N. (2014). New frontiers in the evolution of fin development. J. Exp. Zoology Part B Mol. Dev. Evol. 322 (7), 540–552. 10.1002/jez.b.22563 24677573

[B40] Gai Z. Zhu M. (2012). The origin of the vertebrate jaw: intersection between developmental biology-based model and fossil evidence. Chin. Sci. Bull. 57, 3819–3828. 10.1007/s11434-012-5372-z

[B41] Gai Z. Li Q. Ferrón H. G. Keating J. N. Wang J. Donoghue P. C. J. (2022). Galeaspid anatomy and the origin of vertebrate paired appendages. Nature 609, 959–963. 10.1038/s41586-022-04897-6 36171376

[B42] Garcia Abreu J. Coffinier C. Larraín J. Oelgeschläger M. De Robertis E. M. (2002). Chordin-like CR domains and the regulation of evolutionarily conserved extracellular signaling systems. Gene 287 (1-2), 39–47. 10.1016/s0378-1119(01)00827-7 11992721

[B43] Geetha-Loganathan P. Nimmagadda S. Scaal M. (2008). Wnt signaling in limb organogenesis. Organogenesis 4 (2), 109–115. 10.4161/org.4.2.5857 19279722 PMC2634256

[B44] Gegenbaur C. (1878). *Grundzüge der vergleichenden Anatomie*. Leipzig: Wilhelm Engelmann.

[B45] Gillis J. A. Dahn R. D. Shubin N. H. (2009). Shared developmental mechanisms pattern the vertebrate gill arch and paired fin skeletons. Proc. Natl. Acad. Sci. U. S. A. 106 (14), 5720–5724. 10.1073/pnas.0810959106 19321424 PMC2667079

[B46] Gillis J. A. Modrell M. S. Baker C. V. (2013). Developmental evidence for serial homology of the vertebrate jaw and gill arch skeleton. Nat. Commun. 4, 1436. 10.1038/ncomms2429 23385581 PMC3600657

[B47] Ginzburg A. S. Detlaf T. A. (1975). Sturgeon *Acipenser gueldendstaedti*. Objects of developmental biology. M. Sci., 217–263. (in Russian).

[B48] Glotzer G. L. Tardivo P. Tanaka E. M. (2022). Canonical Wnt signaling and the regulation of divergent mesenchymal Fgf8 expression in axolotl limb development and regeneration. eLife 11, e79762. 10.7554/eLife.79762 35587651 PMC9154742

[B49] Grandel H. Schulte-Merker S. (1998). The development of the paired fins in the zebrafish (*Danio rerio*). Mech. Dev. 79 (1–2), 99–120. 10.1016/s0925-4773(98)00176-2 10349624

[B50] Han J. Young J. W. Frausto R. F. Isenberg S. J. Aldave A. J. (2015). X-linked megalocornea associated with the novel CHRDL1 gene mutation p.(Pro56Leu*8). Ophthalmic Genet. 36 (2), 145–148. 10.3109/13816810.2013.837187 24073597 PMC3968246

[B51] Hawkins M. B. Henke K. Harris M. P. (2021). Latent developmental potential to form limb-like skeletal structures in zebrafish. Cell 184 (4), 899–911.e13. 10.1016/j.cell.2021.01.003 33545089

[B52] Hawkins M. B. Jandzik D. Tulenko F. J. Cass A. N. Nakamura T. Shubin N. H. (2022). An Fgf-Shh positive feedback loop drives growth in developing unpaired fins. Proc. Natl. Acad. Sci. U. S. A. 119 (10), e2120150119. 10.1073/pnas.2120150119 35238632 PMC8916008

[B53] Hikasa H. Ezan J. Itoh K. Li X. Klymkowsky M. W. Sokol S. Y. (2010). Regulation of TCF3 by Wnt-dependent phosphorylation during vertebrate axis specification. Dev. Cell 19 (4), 521–532. 10.1016/j.devcel.2010.09.005 20951344 PMC2963175

[B54] Hiscock T. W. Tschopp P. Tabin C. J. (2017). On the formation of digits and joints during limb development. Dev. Cell 41 (5), 459–465. 10.1016/j.devcel.2017.04.021 28586643 PMC5546220

[B55] Ivanova A. S. Tereshina M. B. Ermakova G. V. Belousov V. V. Zaraisky A. G. (2013). Agr genes, missing in amniotes, are involved in the body appendages regeneration in frog tadpoles. Sci. Rep. 3, 1279. 10.1038/srep01279 23412115 PMC3573343

[B56] Ivanova A. S. Shandarin I. N. Ermakova G. V. Minin A. A. Tereshina M. B. Zaraisky A. G. (2015). The secreted factor Ag1 missing in higher vertebrates regulates fins regeneration in *Danio rerio* . Sci. Rep. 29 (5), 8123. 10.1038/srep08123 25630240 PMC4309956

[B57] Ivanova A. S. Korotkova D. D. Ermakova G. V. Martynova N. Y. Zaraisky A. G. Tereshina M. B. (2018). Ras-dva small GTPases lost during evolution of amniotes regulate regeneration in anamniotes. Sci. Rep. 18 (1), 13035. 10.1038/s41598-018-30811-0 30158598 PMC6115384

[B58] Jin L. Wu J. Bellusci S. Zhang J. S. (2019). Fibroblast growth factor 10 and vertebrate limb development. Front. Genet. 9, 705. 10.3389/fgene.2018.00705 30687387 PMC6338048

[B59] Kariyayama H. Gogoleva N. Harada K. Yokoyama H. Ono H. Suzuki D. G. (2024). Development of the vertebra and fin skeleton in the lamprey and its implications for the homology of vertebrate vertebrae. Dev. Dyn. 253 (3), 283–295. 10.1002/dvdy.657 37732630

[B60] Kawakami Y. Capdevila J. Buscher D. Itoh T. Rodriguez-Esteban C. Izpisua Belmonte J. C. (2001). WNT signals control FGF-dependent limb initiation and AER induction in the chick embryo. Cell 104, 891–900. 10.1016/s0092-8674(01)00285-9 11290326

[B61] Korotkova D. D. Lyubetsky V. A. Ivanova A. S. Rubanov L. I. Seliverstov A. V. Zverkov O. A. (2019). Bioinformatics screening of genes specific for well-regenerating vertebrates reveals c-answer, a regulator of brain development and regeneration. Cell Rep. 29 (4), 1027–1040.e6. 10.1016/j.celrep.2019.09.038 31644900 PMC6871517

[B62] Larraín J. Bachiller D. Lu B. Agius E. Piccolo S. De Robertis E. M. (2000). BMP-binding modules in chordin: a model for signalling regulation in the extracellular space. Development 127 (4), 821–830. 10.1242/dev.127.4.821 10648240 PMC2280033

[B63] Letelier J. Naranjo S. Sospedra-Arrufat I. Martinez-Morales J. R. Lopez-Rios J. Shubin N. (2021). The Shh/Gli3 gene regulatory network precedes the origin of paired fins and reveals the deep homology between distal fins and digits. Proc. Natl. Acad. Sci. U. S. A. 118 (46), e2100575118. 10.1073/pnas.2100575118 34750251 PMC8673081

[B64] Lovely A. M. Duerr T. J. Qiu Q. Galvan S. Voss S. R. Monaghan J. R. (2022). Wnt signaling coordinates the expression of limb patterning genes during axolotl forelimb development and regeneration. Front. Cell Dev. Biol. 10, 814250. 10.3389/fcell.2022.814250 35531102 PMC9068880

[B65] Lyubetsky V. A. Rubanov L. I. Tereshina M. B. Ivanova A. S. Araslanova K. R. Uroshlev L. A. (2023). Wide-scale identification of novel/eliminated genes responsible for evolutionary transformations. Biol. Direct 18 (1), 45. 10.1186/s13062-023-00405-6 37568147 PMC10416458

[B66] Mateus R. Holtzer L. Seum C. Hadjivasiliou Z. Dubois M. Jülicher F. (2020). BMP signaling gradient scaling in the zebrafish pectoral fin. Cell Rep. 30 (12), 4292–4302.e7. 10.1016/j.celrep.2020.03.024 32209485 PMC7109522

[B113] McQueen C. Towers M. (2020). Establishing the pattern of the vertebrate limb. Development. 147 (17), dev177956. 10.1242/dev.177956 32917670

[B67] Mercader N. (2007). Early steps of paired fin development in zebrafish compared with tetrapod limb development. Dev. Growth and Differ. 49, 421–437. 10.1111/j.1440-169X.2007.00942.x 17587327

[B68] Mercader N. Fischer S. Neumann C. J. (2006). Prdm1 acts downstream of a sequential RA, Wnt and Fgf signaling cascade during zebrafish forelimb induction. Development 133, 2805–2815. 10.1242/dev.02455 16790478

[B69] Mivart S. G. (1879). Xii. Notes on the fins of‘ elasmobranchs, with considerations on the nature and homologues of vertebrate limbs. Trans. Zoological Soc. Lond. 10, 439–484. 10.1111/j.1096-3642.1879.tb00460.x

[B70] Miyamoto K. Kawakami K. Tamura K. Abe G. (2022). Developmental independence of median fins from the larval fin fold revises their evolutionary origin. Sci. Rep. 12 (1), 7521. 10.1038/s41598-022-11180-1 35525860 PMC9079066

[B71] Montero J. A. Lorda-Diez C. I. Francisco-Morcillo J. Chimal-Monroy J. Garcia-Porrero J. A. Hurle J. M. (2017). Sox9 expression in amniotes: species-specific differences in the formation of digits. Front. Cell Dev. Biol. 5, 23. 10.3389/fcell.2017.00023 28386540 PMC5362607

[B72] Nakayama N. Han C. E. Scully S. Nishinakamura R. He C. Zeni L. (2001). A novel chordin-like protein inhibitor for bone morphogenetic proteins expressed preferentially in mesenchymal cell lineages. Dev. Biol. 232 (2), 372–387. 10.1006/dbio.2001.0200 11401399

[B73] Nakayama N. Han C. Y. Cam L. Lee J. I. Pretorius J. Fisher S. (2004). A novel chordin-like BMP inhibitor, CHL2, expressed preferentially in chondrocytes of developing cartilage and osteoarthritic joint cartilage. Development 131 (1), 229–240. 10.1242/dev.00901 14660436

[B74] Nieuwkoop P. D. Faber J. (1994). Normal table of *Xenopus laevis* (daudin): a systematical and chronological survey of the development from the fertilized egg till the end of metamorphosis. Garl. Pub. 10.1201/9781003064565

[B75] Onimaru K. Marcon L. Musy M. Tanaka M. Sharpe J. (2016). The fin-to-limb transition as the re-organization of a Turing pattern. Nat. Commun. 7, 11582. 10.1038/ncomms11582 27211489 PMC4879262

[B76] Orlov E. E. Nesterenko A. M. Korotkova D. D. Parshina E. A. Martynova N. Y. Zaraisky A. G. (2022). Targeted search for scaling genes reveals matrix metalloproteinase 3 as a scaler of the dorsal-ventral pattern in *Xenopus laevis* embryos. Dev. Cell 57 (1), 95–111.e12. 10.1016/j.devcel.2021.11.021 34919801

[B77] Pascual-Anaya J. D’Aniello S. Bertrand S. (2022). Editorial: new approaches in chordate and vertebrate evolution and development. Front. Cell Dev. Biol. 10, 917101. 10.3389/fcell.2022.917101 35646936 PMC9134185

[B78] Pfirrmann T. Emmerich D. Ruokonen P. Quandt D. Buchen R. Fischer-Zirnsak B. (2015). Molecular mechanism of CHRDL1-mediated X-linked megalocornea in humans and in Xenopus model. Hum. Mol. Genet. 24 (11), 3119–3132. 10.1093/hmg/ddv063 25712132

[B79] Piccolo S. Agius E. Lu B. Goodman S. Dale L. De Robertis E. M. (1997). Cleavage of Chordin by Xolloid metalloprotease suggests a role for proteolytic processing in the regulation of Spemann organizer activity. Cell 91 (3), 407–416. 10.1016/s0092-8674(00)80424-9 9363949 PMC3070600

[B80] Pieretti J. Gehrke A. R. Schneider I. Adachi N. Nakamura T. Shubin N. H. (2015). Organogenesis in deep time: a problem in genomics, development, and paleontology. Proc. Natl. Acad. Sci. U. S. A. 112 (16), 4871–4876. 10.1073/pnas.1403665112 25901307 PMC4413340

[B81] Pierreux C. E. Nicolás F. J. Hill C. S. (2000). Transforming growth factor beta-independent shuttling of Smad4 between the cytoplasm and nucleus. Mol. Cell Biol. 20 (23), 9041–9054. 10.1128/MCB.20.23.9041-9054.2000 11074002 PMC86557

[B82] Pizette S. Abate-Shen C. Niswander L. (2001). BMP controls proximodistal outgrowth, via induction of the apical ectodermal ridge, and dorsoventral patterning in the vertebrate limb. Development 128 (22), 4463–4474. 10.1242/dev.128.22.4463 11714672

[B83] Plouhinec J. L. Zakin L. Moriyama Y. De Robertis E. M. (2013). Chordin forms a self-organizing morphogen gradient in the extracellular space between ectoderm and mesoderm in the Xenopus embryo. Proc. Natl. Acad. Sci. U. S. A. 110 (51), 20372–20379. 10.1073/pnas.1319745110 24284174 PMC3870759

[B84] Pradel A. Maisey J. G. Tafforeau P. Mapes R. H. Mallatt J. (2014). A Palaeozoic shark with osteichthyan-like branchial arches. Nature 509 (7502), 608–611. 10.1038/nature13195 24739974

[B85] Raspopovic J. Marcon L. Russo L. Sharpe J. (2014). Modeling digits: digit patterning is controlled by a Bmp-Sox9-Wnt Turing network modulated by morphogen gradients. Science 345 (6196), 566–570. 10.1126/science.1252960 25082703

[B86] Riddle R. D. Johnson R. L. Laufer E. Tabin C. (1993). Sonic hedgehog mediates the polarizing activity of the ZPA. Cell 75 (7), 1401–1416. 10.1016/0092-8674(93)90626-2 8269518

[B87] Sakuta H. Suzuki R. Takahashi H. Kato A. Shintani T. Iemura S. (2001). Ventroptin: a BMP-4 antagonist expressed in a double-gradient pattern in the retina. Science 293 (5527), 111–115. 10.1126/science.1058379 11441185

[B88] Satoh A. Suzuki M. Amano T. Tamura K. Ide H. (2005). Joint development in *Xenopus laevis* and induction of segmentations in regenerating froglet limb (spike). Dev. Dyn. 233 (4), 1444–1453. 10.1002/dvdy.20484 15977182

[B89] Schmidt J. Francois V. Bier E. Kimelman D. (1995). Drosophila short gastrulation induces an ectopic axis in Xenopus: evidence for conserved mechanisms of dorsal-ventral patterning. Development 121 (12), 4319–4328. 10.1242/dev.121.12.4319 8575332

[B115] Sheeba C. J. Andrade R. P. Palmeirim I. (2016). Getting a handle on embryo limb development: Molecular interactions driving limb outgrowth and patterning. Semin. Cell Biol. 49, 92–101. 10.1016/j.semcdb.2015.01.007 25617599

[B90] Shimada A. Kawanishi T. Kaneko T. Yoshihara H. Yano T. Inohaya K. (2013). Trunk exoskeleton in teleosts is mesodermal in origin. Nat. Commun. 4, 1639. 10.1038/ncomms2643 23535660 PMC3615485

[B91] Shkil F. Kapitanova D. Borisov V. Veretennikov N. Roux N. Laudet V. (2022). Direct development of the catfish pectoral fin: an alternative pectoral fin pattern of teleosts. Dev. Dyn. 251 (11), 1816–1833. 10.1002/dvdy.509 35706124

[B92] Shmalgauzen O. I. (1975). Sturgeon *Acipenser gueldenstaedti colchicus*. Development of prelarvae. Objects of developmental biology. Science, 264–277. (in Russian).

[B93] Sive H. Grainger R. Harland R. (2000). Early development of *Xenopus laevis*: a laboratory manual. Long Island, New York: Cold Spring Harbor Laboratory Press.

[B94] Sleight V. A. Gillis J. A. (2020). Embryonic origin and serial homology of gill arches and paired fins in the skate, *Leucoraja erinacea* . eLife 9, e60635. 10.7554/eLife.60635 33198887 PMC7671686

[B95] Striedter G. F. Northcutt R. G. (2020). “The origin of jaws and paired fins: the age of fishes,” in *Brains through time: a natural History of vertebrates* (online edn) (Oxford Academic). 10.1093/oso/9780195125689.003.0003

[B96] Sun X. Mariani F. V. Martin G. R. (2002). Functions of FGF signalling from the apical ectodermal ridge in limb development. Nature 418 (6897), 501–508. 10.1038/nature00902 12152071

[B97] Tanaka Y. Miura H. Tamura K. Abe G. (2022). Morphological evolution and diversity of pectoral fin skeletons in teleosts. Zool. Lett. 8 (1), 13. 10.1186/s40851-022-00198-y PMC9701400 36435818

[B99] Ten Berge D. Koole W. Fuerer C. Fish M. Eroglu E. Nusse R. (2008). Wnt signaling mediates self-organization and axis formation in embryoid bodies. Cell Stem Cell 3 (5), 508–518. 10.1016/j.stem.2008.09.013 18983966 PMC2683270

[B100] Thacher J. K. (1877). Median and paired fins, a contribution to the history of vertebrate limbs. Trans. Conn Acad. Arts Sci. 3, 281–308.

[B101] Thompson A. W. Hawkins M. B. Parey E. Wcisel D. J. Ota T. Kawasaki K. (2021). The bowfin genome illuminates the developmental evolution of ray-finned fishes. Nat. Genet. 53, 1373–1384. 10.1038/s41588-021-00914-y 34462605 PMC8423624

[B102] Tickle C. Towers M. (2017). Sonic Hedgehog signaling in limb development. Front. Cell Dev. Biol. 5, 14. 10.3389/fcell.2017.00014 28293554 PMC5328949

[B103] Troilo H. Zuk A. V. Tunnicliffe R. B. Wohl A. P. Berry R. Collins R. F. (2014). Nanoscale structure of the BMP antagonist chordin supports cooperative BMP binding. Proc. Natl. Acad. Sci. U. S. A. 111 (36), 13063–13068. 10.1073/pnas.1404166111 25157165 PMC4246984

[B104] Tulenko F. J. Massey J. L. Holmquist E. Kigundu G. Thomas S. Smith S. M. E. (2017). Fin-fold development in paddlefish and catshark and implications for the evolution of the autopod. Proc. Biol. Sci. 284 (1855), 20162780. 10.1098/rspb.2016.2780 28539509 PMC5454254

[B106] Webb T. R. Matarin M. Gardner J. C. Kelberman D. Hassan H. Ang W. (2012). X-linked megalocornea caused by mutations in CHRDL1 identifies an essential role for ventroptin in anterior segment development. Am. J. Hum. Genet. 90 (2), 247–259. 10.1016/j.ajhg.2011.12.019 22284829 PMC3276677

[B107] Yano T. Abe G. Yokoyama H. Kawakami K. Tamura K. (2012). Mechanism of pectoral fin outgrowth in zebrafish development. Development 139 (22), 2916–2925. 10.1242/dev.075572 22791899

[B109] Yonei-Tamura S. Abe G. Tanaka Y. Anno H. Noro M. Ide H. (2008). Competent stripes for diverse positions of limbs/fins in gnathostome embryos. Evol. Dev. 10 (6), 737–745. 10.1111/j.1525-142X.2008.00288.x 19021745

[B110] Zaraisky A. G. Araslanova K. R. Shitikov A. D. Tereshina M. B. (2024). Loss of the ability to regenerate body appendages in vertebrates: from side effects of evolutionary innovations to gene loss. Biol. Rev. Camb Philos. Soc. 99 (5), 1868–1888. 10.1111/brv.13102 38817123

[B111] Zeller R. López-Ríos J. Zuniga A. (2009). Vertebrate limb bud development: moving towards integrative analysis of organogenesis. Nat. Rev. Genet. 10 (12), 845–858. 10.1038/nrg2681 19920852

[B112] Zhang X. Shimoda K. Ura K. Adachi S. Takagi Y. (2012). Developmental structure of the vertebral column, fins, scutes and scales in bester sturgeon, a hybrid of beluga *Huso huso* and sterlet *Acipenser ruthenus* . J. Fish. Biol. 81 (6), 1985–2004. 10.1111/j.1095-8649.2012.03451.x 23130694

