# Supplementary figures and images for: The emergence of chordin-like1 in gnathostomes may have contributed to the evolution of paired appendages
Source: Front Cell Dev Biol. 2025 Aug 29;13:1649996. doi: 10.3389/fcell.2025.1649996 (PMC12425909; doi:10.3389/fcell.2025.1649996)

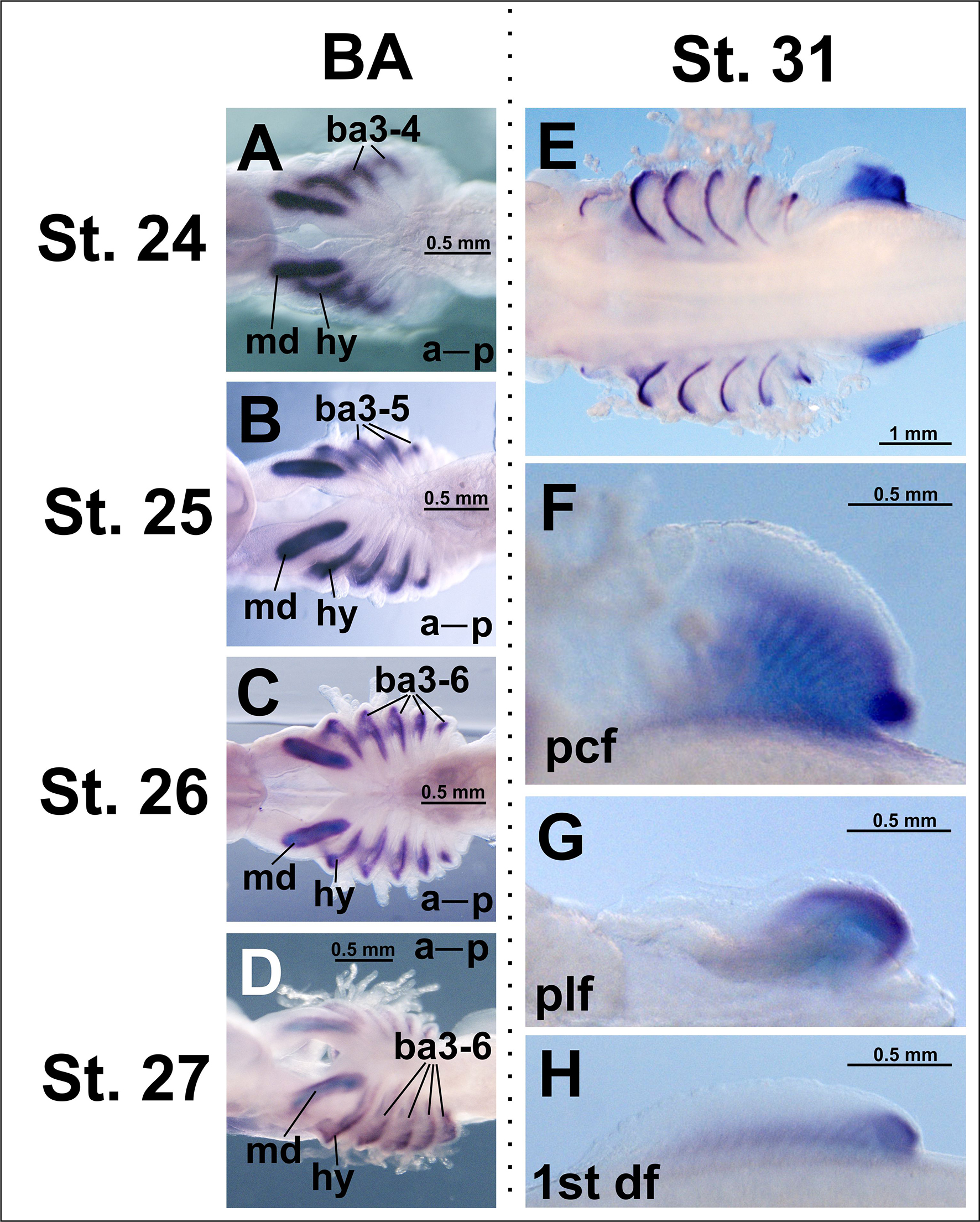

Supplement: Supplementary file 1 [file Image3.jpeg]

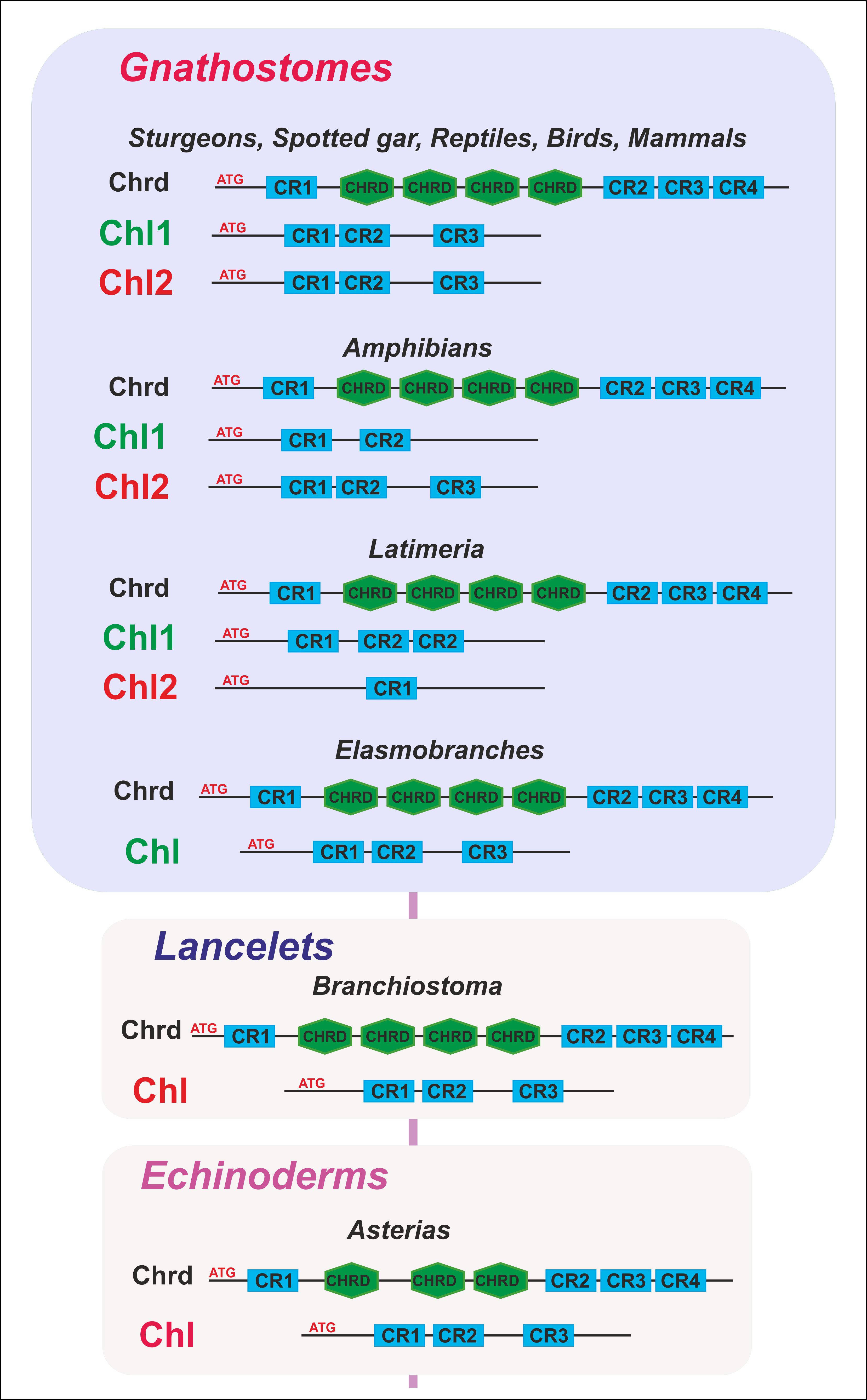

Supplement: Supplementary file 2 [file Image1.jpeg]

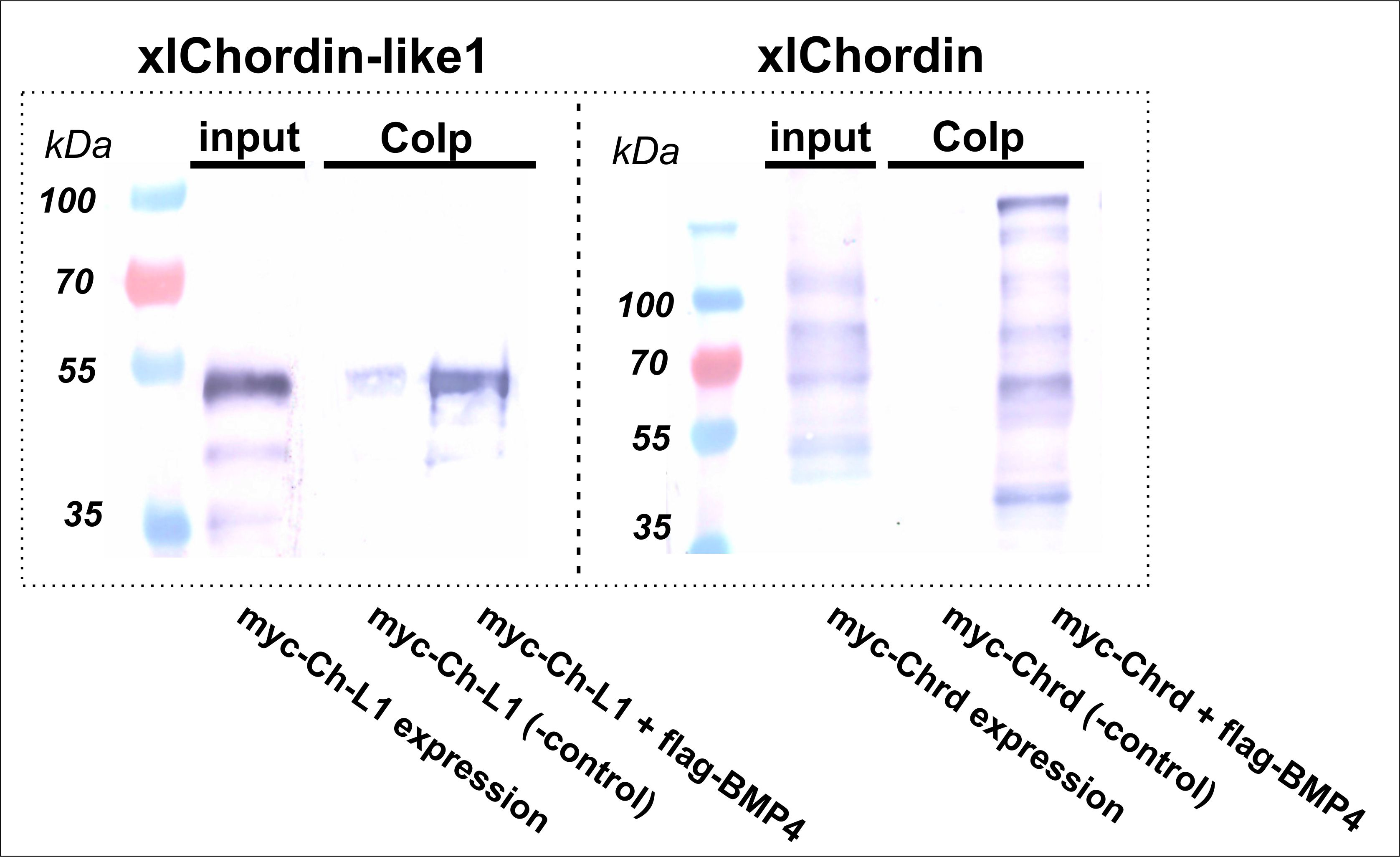

Supplement: Supplementary file 3 [file Image4.jpeg]

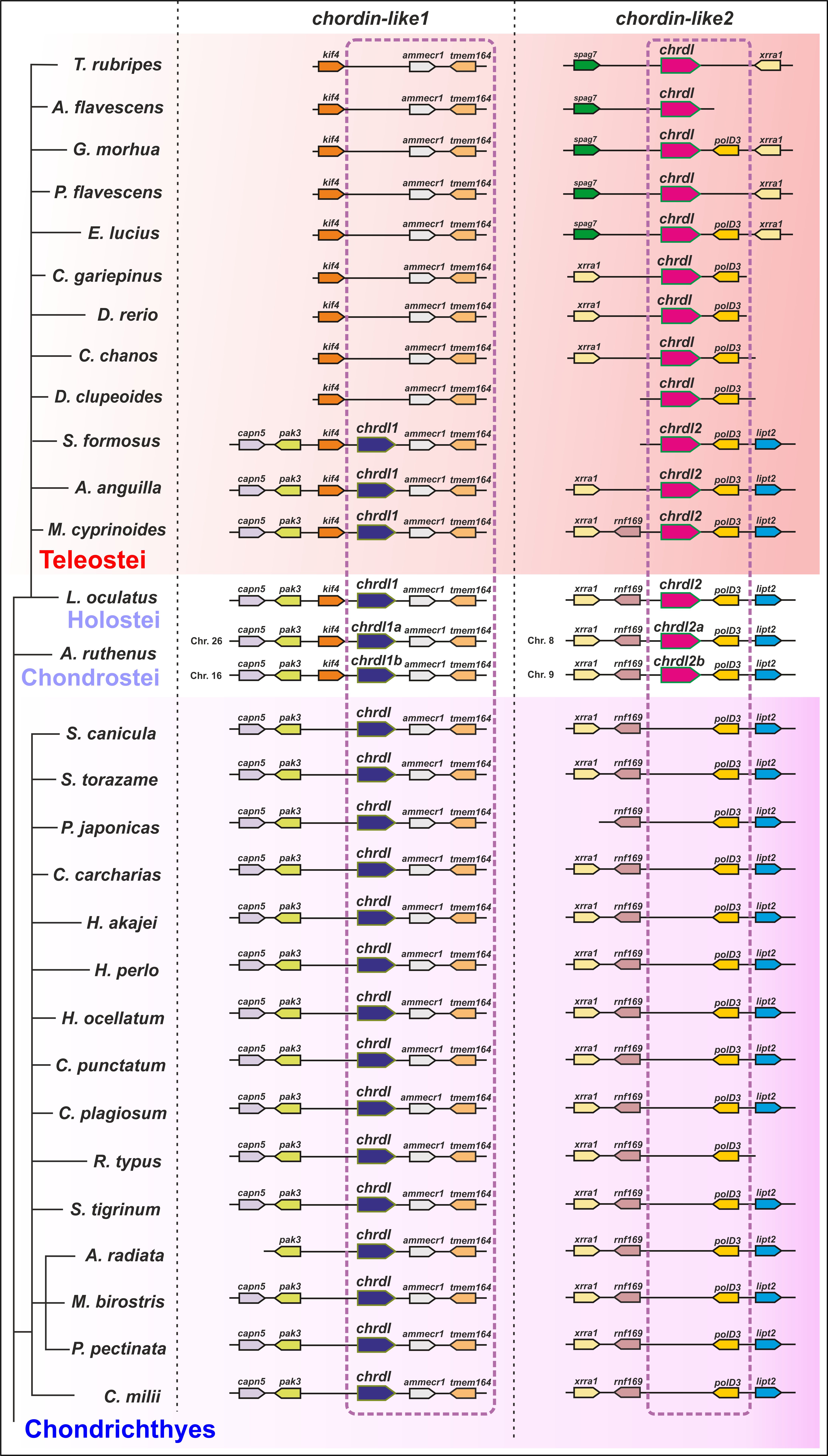

Supplement: Supplementary file 4 [file Image2.jpeg]
